# Cell-type dependent enhancer binding of the EWS/ATF1 fusion gene in clear cell sarcomas

**DOI:** 10.1038/s41467-019-11745-1

**Published:** 2019-09-05

**Authors:** Shingo Komura, Kenji Ito, Sho Ohta, Tomoyo Ukai, Mio Kabata, Fumiaki Itakura, Katsunori Semi, Yutaka Matsuda, Kyoichi Hashimoto, Hirofumi Shibata, Masamitsu Sone, Norihide Jo, Kazuya Sekiguchi, Takatoshi Ohno, Haruhiko Akiyama, Katsuji Shimizu, Knut Woltjen, Manabu Ozawa, Junya Toguchida, Takuya Yamamoto, Yasuhiro Yamada

**Affiliations:** 1 Center for iPS Cell Research and Application (CiRA), Kyoto University, Kyoto, 606-8507 Japan; 2 Department of Orthopaedic Surgery, Gifu University Graduate School of Medicine, Gifu, 501-1194 Japan; 3 Division of Stem Cell Pathology, Center for Experimental Medicine and Systems Biology, Institute of Medical Science, University of Tokyo, Tokyo, 108-8639 Japan; 4 Research Division, Chugai Pharmaceutical Co., Ltd, Kamakura, Japan; 5 Department of Tissue Regeneration, Institute for Frontier Medical Sciences, Kyoto University, Kyoto, 606-8507 Japan; 6 Department of Orthopaedic Surgery, Graduate School of Medicine, Kyoto University, Kyoto, 606-8507 Japan; 7 Spine Center, Gifu Municipal Hospital, Gifu, 500-8513 Japan; 8 Hakubi Center for Advanced Research, Kyoto University, Kyoto, 606-8501 Japan; 9 Laboratory of Reproductive Systems Biology, Center for Experimental Medicine and Systems Biology, Institute of Medical Science, University of Tokyo, Tokyo, 108-8639 Japan; 10 Institute for the Advanced Study of Human Biology (WPI-ASHBi), Kyoto University, Yoshida-Konoe-cho, Sakyo-ku, Kyoto, 606-8501 Japan; 11 Medical-risk Avoidance based on iPS Cells Team, RIKEN Center for Advanced Intelligence Project (AIP), Kyoto, 606-8507 Japan; 12 AMED-CREST, AMED 1-7-1 Otem ach, Chiyodaku, Tokyo, 100-0004 Japan

**Keywords:** Cancer models, Reprogramming

## Abstract

Clear cell sarcoma (CCS) is a rare soft tissue sarcoma caused by the EWS/ATF1 fusion gene. Here, we established induced pluripotent stem cells (iPSCs) from *EWS/ATF1*-controllable murine CCS cells harboring sarcoma-associated genetic abnormalities. Sarcoma-iPSC mice develop secondary sarcomas immediately after EWS/ATF1 induction, but only in soft tissue. *EWS/ATF1* expression induces oncogene-induced senescence in most cell types in sarcoma-iPSC mice but prevents it in sarcoma cells. We identify *Tppp3*-expressing cells in peripheral nerves as a cell-of-origin for these sarcomas. We show cell type-specific recruitment of EWS/ATF1 to enhancer regions in CCS cells. Finally, epigenetic silencing at these enhancers induces senescence and inhibits CCS cell growth through altered EWS/ATF1 binding. Together, we propose that distinct responses to premature senescence are the basis for the cell type-specificity of cancer development.

## Abstract (web summary)

The EWS-ATF1 fusion gene causes clear cell sarcoma (CCS). Here, the authors show that the downstream effects of EWS-ATF1 expression are strictly context dependent, and reveal the cell of origin for CCS to be Tppp3-expressing cells in peripheral nerves.

## Introduction

Cell-type-specific development of cancer has long been recognized. Here, specific genes are mutated in particular types of cancer, and patients harboring germline mutations at tumor suppressor genes, such as *APC* and *BRCA1/BRCA2*, exclusively develop cancers in specific organs^1–3^. Similarly, differentiation status-dependent cancer development has been reported in several cancers^4,5^. Given that epigenetic regulations play a central role in differentiation and cell fate maintenance, these findings suggest that a coordinated action of the cancer genome and cellular context-dependent epigenetic regulations is required for cancer development. However, it is unclear how the cellular context-dependent epigenetic regulations affect cancer development and progression.

Clear-cell sarcoma (CCS) is a rare tumor and preferentially arises in soft tissue in the extremities. CCS often harbors melanocytic properties, and thus resembles malignant melanoma^6,7^. Despite their shared properties, CCS is distinguished from malignant melanoma by the presence of a specific fusion oncogene, *EWS/ATF1*, which results from a reciprocal translocation, t(12;22)(q13;q12)^8^. Previous in vivo studies using *EWS/ATF1*-inducible CCS model demonstrated that *EWS/ATF1* is a key driver oncogene in both the development and maintenance of the sarcomas^9,10^. Straessler et al.^10^ demonstrated that *EWS/ATF1* can transform mesenchymal progenitor cells^10^, while our previous study suggested that the sarcomas arose from neural crest-derived cells^9^. However, given that mesenchymal progenitor cells and neural crest-derived cells contain diverse cell types, a cell of origin for this sarcoma remains to be fully understood.

In the present study, we establish induced pluripotent stem cells (iPSCs) from murine CCS cell lines that harbor sarcoma-associated genetic abnormalities and differentiate them into a wide variety of cell types in vivo via the generation of chimeric mice. The chimeric mice rapidly develop secondary sarcomas in a cell-type-dependent manner, which is associated with a cell-type-specific response to premature senescence. Utilizing the one-step and cell-type-specific sarcoma model, we identify *Tppp3*-expressing peripheral nerve cell as a cell type that gives rise to CCSs and demonstrate that cellular context-dependent epigenetic regulations, in conjunction with genetic abnormalities, play a fundamental role in the maintenance of the malignant phenotype and thus can be a therapeutic target in sarcoma cells.

## Results

### Establishment of iPSCs from *EWS/ATF1*-induced sarcoma cells

**Fig. 1:**
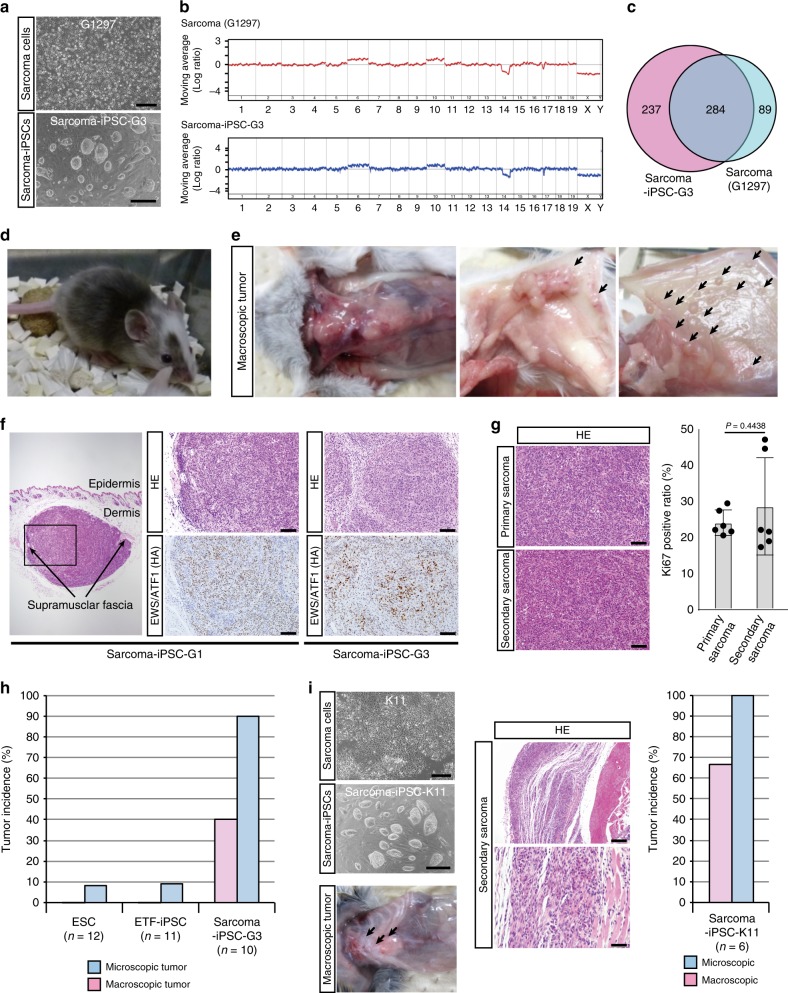
One-step secondary sarcoma development in sarcoma-induced pluripotent stem cells (iPSC) mice. **a** iPSC-like cells were generated from a murine clear-cell sarcoma (CCS) cell line (G1297) by introducing four reprogramming factors. Scale bars: 600 µm (upper), 300 µm (lower). **b** Array comparative genomic hybridization (CGH) analysis reveals that sarcoma-derived iPSC-like cells (clone G3) share identical chromosomal aberrations with parental sarcoma cells (G1297). **c** Candidate mutation sites in sarcoma cells (G1297) and sarcoma-derived iPSC-like cells (clone G3). Exome analysis reveals that more than 75% of candidate mutation sites in G1297 are overlapped with those in sarcoma-derived iPSC-like cells. **d** Sarcoma-derived iPSC-like cells contributed to adult chimeric mice after injection into blastocyst. **e** Secondary sarcoma development in sarcoma-iPSC mice treated with doxycycline (Dox). Secondary sarcoma preferentially arose in soft tissue. Left, center of chest and abdominal wall (Dox 1 week). Middle, subcutaneous tissue; right, abdominal fascia (Dox 4–5 weeks). **f** Secondary sarcoma developed at the subcutaneous layer (left) in sarcoma-iPSC mouse. Both iPSC G1- and G3-derived mice developed subcutaneous tumors. Histological analysis reveals that secondary sarcomas resemble primary CCS. EWS/ATF1 expression is detectable in secondary tumors by immunohistochemistry using hemagglutinin (HA) antibody. Scale bars: 100 µm. **g** Histology and Ki67-positive cell ratio of primary CCSs and secondary sarcomas. Scale bars: 50 µm. Primary CCSs were obtained from *EWS/ATF1*-induced embryonic stem cell (ESC) mice (*Rosa-M2rtTA*/*Col1a1::tetO-EWS/ATF1-ires-mCherry*), while secondary sarcomas were obtained from sarcoma-iPSC mice, which were generated with two independent iPSC clones (G1 and G3). The mean ± SD of six independent primary and secondary sarcomas are shown, respectively. No significant difference in the Ki67-positive cell ratio is observed between them (*P* = 0.4438, two-sided Student’s *t* test). **h** Tumor incidence within 2 weeks after Dox administration in chimeric mice generated with control ESCs, ETF-iPSCs (ear-tip fibroblast-iPSCs), and sarcoma-iPSCs. Sarcoma-iPSC mice developed secondary sarcomas at a higher incidence and shorter latency than other mice. Pink bar shows the incidence of macroscopic sarcoma, and blue bar shows the incidence of microscopic sarcoma. **i** iPSC derivation from an independent sarcoma cell line (K11). A higher incidence and shorter latency of secondary sarcoma development were observed in sarcoma (K11)-iPSC mice within 2 weeks. Scale bars: 600 µm (left upper), 300 µm (left lower), 200 µm (right upper), and 50 µm (right lower)

To establish iPSCs, we introduced *OCT3/4*, *SOX2*, *KLF4* and *cMYC* into a murine CCS cell line (G1297) containing doxycycline (Dox)-controllable *EWS/ATF1* alleles^9^. iPSC-like colonies were picked up to establish iPSC-like cell lines (Fig. 1a). The established iPSC-like clones showed elevated activity of alkaline phosphatase and expressed pluripotency-related genes such as *Nanog* and endogenous *Oct3/4* (Supplementary Fig. 1a, b). Global gene expression patterns of iPSC-like cells were similar to those of control PSCs (Supplementary Fig. 1c, d). DNA methylation analysis revealed demethylation of *Nanog* promoter and of *Oct3/4* distal enhancer in iPSC-like cells (Supplementary Fig. 1e). Silencing of the exogenous 4 factors was confirmed in most iPSC-like clones (Supplementary Fig. 1f). The iPSC-like cell lines often harbored the chromosomal abnormalities detected in G1297 (Fig. 1b and Supplementary Fig. 1g). Additionally, exome analysis revealed that candidate mutation sites in G1297 are often mutated in iPSC-like cell line (Sarcoma-iPSC-G3) (Fig. 1c, Supplementary Fig. 1h, and Supplementary Table 1). Three independent sarcoma-iPSC-like clones (G1, G2, and G3) formed teratomas and contributed to adult chimeric mice (Supplementary Fig. 1i and Fig. 1d). Together, these results confirm the establishment of iPSCs that harbor cancer-related genetic abnormalities from mouse CCS cells in the absence of *EWS/ATF1* expression.

### Secondary sarcoma development in sarcoma-iPSC mice

We next examined secondary sarcoma development^11,12^ in sarcoma-iPSC chimeric mice by inducing *EWS/ATF1*. For control experiments, chimeric mice were generated with embryonic stem cells (ESCs) and iPSCs, both of which contain *EWS/ATF1*-inducible alleles at the identical loci as sarcoma-iPSCs (Supplementary Fig. 2a, b). We confirmed that sarcoma-derived iPSCs expressed similar levels of *EWS/ATF1* with control ESCs and ETF-iPSCs (ear-tip fibroblast-iPSCs) upon Dox exposure (Supplementary Fig. 2c).

Notably, upon Dox administration, sarcoma-iPSC chimeric mice developed sarcomas much earlier than ESC- and ETF-iPSC-derived chimeric mice (Fig. 1e, f and Table 1). Histological features of secondary sarcomas in the sarcoma-iPSC mice were similar to those of both primary sarcomas and human CCSs (Fig. 1g). Furthermore, cell proliferative activity was not significantly altered in secondary sarcomas (Fig. 1g and Supplementary Fig. 2d). In sarcoma-iPSC mice, *EWS/ATF1* induction resulted in microscopic sarcoma formation in 90% of mice (9/10) and macroscopic sarcoma development in 40% of mice (4/10) within 2 weeks (Fig. 1h and Table 1). In sharp contrast, 2 weeks treatment with Dox for both ESC-derived and ETF-iPSC-derived chimeric mice resulted in no macroscopic sarcoma formation (0/12 and 0/11, respectively), and only a few mice developed microscopic sarcomas (1/12 in ESC mice and 1/11 in ETF-iPSC mice) (Fig. 1h and Table 1). These results are consistent with a previous melanoma cloning study by somatic cell nuclear transfer, in which melanoma-derived chimeric mice developed secondary melanomas with shorter latency^13^. In contrast, sarcoma-iPSC mice that were not given Dox were vigorous and did not develop any CCS-like sarcomas even at 6 months of age (*n* = 7), except for one mouse that developed *EWS/ATF1*-independent rhabdomyosarcoma (Supplementary Fig. 2e, f). We also established an iPSC line (sarcoma-iPSC-K11) from an independent sarcoma cell line (K11) and confirmed the rapid secondary sarcoma development in the sarcoma-iPSC chimeric mice (Fig. 1i and Supplementary Fig. 2g). Collectively, we established a one-step in vivo cancer model using sarcoma-derived iPSCs.

**Table 1:** Tumor development after induction of *EWS/ATF1*

	Coat color H: >60%; M: 30–60%; L: <30%	*EWS/ATF1* induction (weeks)	Microscopic tumor	Macroscopic tumor	Location of tumors
ESC chimeric mice					
#3–8	H	1	–	–	
#2–7	H	1	–	–	
#2–8	H	1	–	–	
#3–5	H	1	–	–	
#3–6	H	1	–	–	
#3–7	H	1	–	–	
#2–5	H	2	–	–	
#2–9	M	2	–	–	
#2–10	H	2	–	–	
#2–11	M	2	–	–	
#2–14	M	2	–	–	
#2–6	H	2	+	–	Eye
#2–3	L	4	–	–	
#3–3	L	4	–	–	
#3–4	H	4	–	–	
#2–18	L	4	–	–	
#2–19	L	4	–	–	
#2–13	L	4	+	–	Abdominal wall
#2–17	L	4	+	–	Subcutaneous tissue
#2–4	M	4	+	+	Chest wall
#2–12	M	8	+	+	Chest wall
#2–15	M	8	+	+	Chest wall
#2–16	M	8	+	+	Chest wall abdominal wall
#2-1	H	12	–	–	
#2-2	H	12	–	–	
#3-1	L	12	–	–	
#3-2	L	12	–	–	
			7/27	4/27	
ETF-iPSC chimeric mice					
109 #1-1	M	1	–	–	
109 #1–2	M	1	–	–	
109 #1–3	L	1	–	–	
112 #1–4	H	1	–	–	
112 #1–5	M	1	–	–	
112 #1–6	M	1	–	–	
109 #1–7	H	2	–	–	
109 #1–8	M	2	–	–	
109 #1–11	M	2	–	–	
112 #1–2	L	2	–	–	
112 #1-1	M	2	+	–	Eye
109 #1–5	L	4	–	–	
109 #1–6	L	4	–	–	
112 #7-1	M	4	–	–	
109 #1–4	L	4	+	–	Abdominal fascia
112 #1–3	L	4	+	–	Subcutaneous tissue
109 #1–9	L	8	–	–	
109 #1–10	M	8	–	–	
112 #1–8	L	8	–	–	
112 #1–9	L	8	–	–	
112 #1–7	L	8	–	–	
112 #7-2	L	8	–	–	
109 #1–12	L	12	–	–	
109 #1–13	L	12	–	–	
			3/24	0/24	
Sarcoma-iPSC chimeric mice					
#3 6-3	M	1	–	–	
#3 6-2	M	1	+	–	Abdominal wall
#3 4-2	H	1	+	–	Subcutaneous tissue
#3 5-4	M	1	+	–	Abdominal wall
#3 6-1	M	1	+	–	Abdominal wall
#3 3-1	H	1	+	+	Chest wall abdominal wall eye
#3 10-1	H	1	+	+	Abdominal wall eye
#3 5-3	H	1	+	+	Eye subscapula
#3 2-1	H	2	+	–	Peri-rib
#3 6-4	L	2	+	+	Abdominal wall
#1 2-1	L	4	+	+	Eye subcutaneous fascia
1 2-2	L	4	+	+	Eye
#1 1-1	M	8	–	–	
#1 1-3	L	8	–	–	
#3 5-1	M	8	–	–	
#3 6-5	L	8	–	–	
#3 7-1	M	8	–	–	
#1 2-3	L	8	+	+	Eye
#1 1-2	L	8	+	+	Eye subcutaneous tissue gastric wall
#3 5-2	L	8	+	+	Abdominal wall peri-rib eye
#3 7-2	M	8	+	+	Achilles tendon eye abdominal wall mammary gland
#3 1-1	M	12	–	–	
#3 4-3	L	12	–	–	
			15/23	10/23	

*ESC* embryonic stem cell, *ETF* ear-tip fibroblast, *iPSC* induced pluripotent stem cell

### Cell-type-specific development of secondary sarcomas

**Fig. 2:**
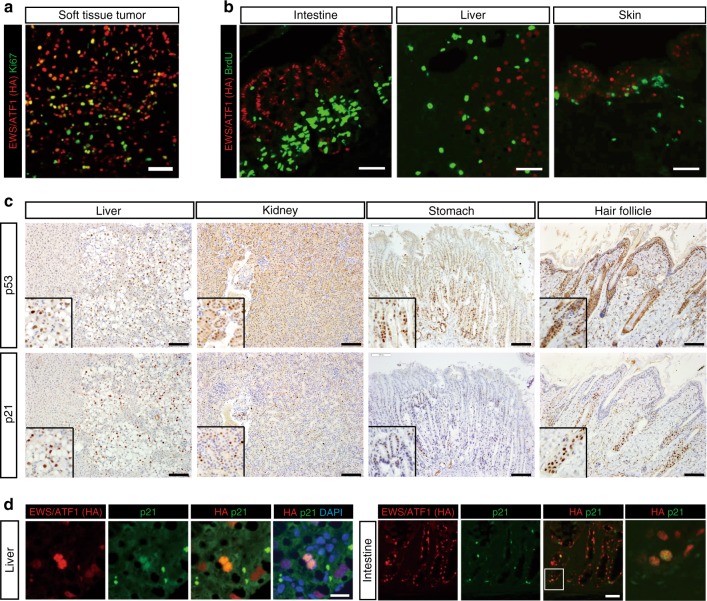
Cellular context-dependent senescence response to *EWS/ATF1* expression. **a** Immunostaining of secondary sarcoma in soft tissue using hemagglutinin (HA) and Ki67 antibodies reveals that EWS/ATF1-expressing cells have high proliferative activity in tumor. Scale bar: 50 µm. **b** Immunofluorescence of several organs and tissues in sarcoma-induced pluripotent stem cell (iPSC) chimeric mice given doxycycline (Dox) for 5 days using HA and bromodeoxyuridine (BrdU) antibodies. HA-positive EWS/ATF1-expressing cells are exclusively negative for BrdU, indicating EWS/ATF1-expressing cells acquired no malignant proliferation. Scale bars: 50 µm. **c** Immunostaining for p53 and p21 in multiple organs of sarcoma-iPSC chimeric mice given Dox for 5 days. Note that p53- and p21-positive cells are frequently observed in the liver, kidney, stomach, and hair follicle. Insets indicate higher magnification. Scale bars: 100 µm. **d** p21-positive cells are often overlapped with EWS/ATF1-expressing somatic cells in sarcoma-iPSC mice. Scale bars: 100 µm

Immunohistochemical analysis revealed that *EWS/ATF1* is expressed in various organs and tissues of sarcoma-iPSC mice after Dox treatment (day 5, Supplementary Fig. 3a). Notably, *EWS/ATF1* expression induced secondary sarcomas preferentially at the soft tissues, such as fascia, subcutaneous tissue, tissue adjacent to the tendon, and soft tissue outside the eyeballs (Figs. 1e, f, 2a and Supplementary Fig. 3b). However, despite *EWS/ATF1* expression in a wide variety of organs, such as the intestine, liver, and skin, *EWS/ATF1* did not induce abnormal proliferation in the cells of these tissues (day 5, Fig. 2b). In contrast, we observed proliferating cells in the soft tissue immediately after Dox treatment (day 5, Supplementary Fig. 3c, d), indicating that the initiation of abnormal growth is dependent on cell type.

We next asked why *EWS/ATF1* expression fails to induce cancer development in most cell types. It has been shown that oncogene expression in somatic cells results in oncogene-induced senescence (OIS), which prevents cancer development^14,15^. First, we examined p53 and p21 expression, a representative OIS marker^16^, in multiple organs of sarcoma-iPSC mice after Dox treatment for 5 days. Notably, an increased number of both p53- and p21-expressing cells was detectable in accordance with cells expressing EWS/ATF1 in multiple organs at day 5 (Fig. 2c, d), suggesting that OIS occurs in most cell types in sarcoma-iPSC mice.

**Fig. 3:**
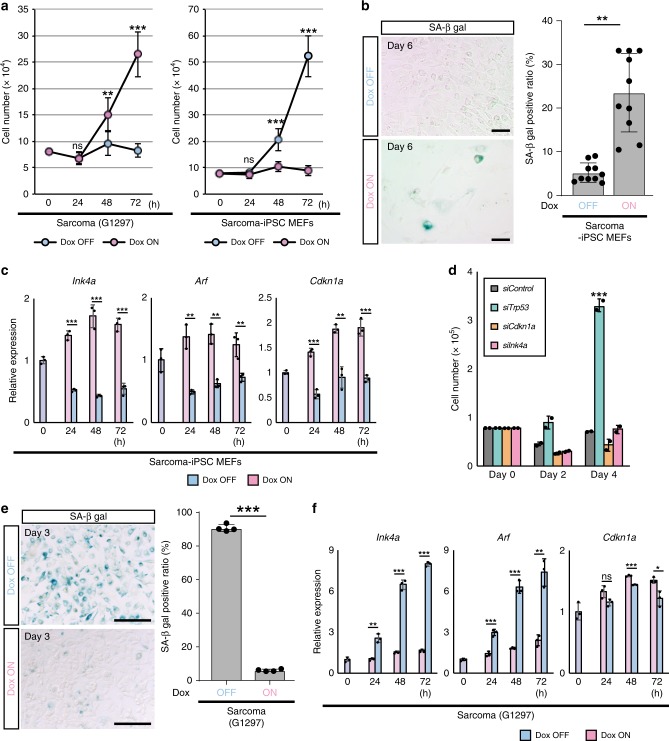
Opposing effects of *EWS/ATF1* on cellular senescence depending on cell type. **a** Sarcoma-induced pluripotent stem cell (iPSC) mouse embryonic fibroblasts (MEFs) cease proliferation in the presence of doxycycline (Dox). In contrast, G1297 proliferate only in the presence of Dox (ns; not significant, **P < 0.01, ***P < 0.001, two-sided Student’s *t* test). Dox concentration is 0.2 µg/ml. The mean ± SD of three independent biological experiments are shown. **b** Representative images of senescence-associated β-galactosidase (SA β-gal) staining of sarcoma-iPSC MEF with/without Dox treatment (0.2 µg/ml, 6 days). SA β-gal-positive cells are frequently observed in the Dox ON condition. SA β-gal-positive ratio of Dox OFF- and Dox ON-sarcoma-iPSC MEFs are shown in the right panel. Frequency of SA β-gal-positive cells significantly increases with *EWS/ATF1* expression (**P < 0.01, two-sided Student’s *t* test). The mean ± SD of 10 independent biological experiments are shown, respectively. Scale bars: 200 µm. **c** A real-time quantitative RT-PCR (qRT-PCR) analysis for *Ink4a*, *Arf*, and *Cdkn1a* in sarcoma-iPSC MEFs after exposure to Dox (**P < 0.01, ***P < 0.001, two-sided Student’s *t* test). Data are presented as the mean ± SD of biological triplicates. The mean expression level of samples at 0 h was set to 1. **d** Knockdown of senescence-related genes in sarcoma-iPSC MEFs. Note that knockdown of *Trp53* results in escape from growth arrest of Dox-treated sarcoma-iPSC MEFs (***P < 0.001, two-sided Student’s *t* test). The mean ± SD of two independent biological experiments are shown, respectively. **e** Representative images of SA β-gal staining of sarcoma cells (G1297) with/without Dox treatment (0.2 µg/ml, 3 days). Note that most Dox OFF cells are positive for SA β-gal. SA β-gal-positive ratio of Dox OFF- and Dox ON-sarcoma cells are shown in the right panel. Frequency of SA β-gal-positive cells significantly increases after withdrawal of *EWS/ATF1* expression (***P < 0.001, two-sided Student’s *t* test). The mean ± SD of four independent biological experiments are shown, respectively. Scale bars: 200 µm. **f** A qRT-PCR analysis for *Ink4a*, *Arf*, and *Cdkn1a* in sarcoma cells after withdrawal of Dox. Sarcoma cells exhibit an increased expression of *Ink4a* and *Arf* upon *EWS/ATF1* withdrawal (ns; not significant, *P < 0.05, **P < 0.01, ***P < 0.001, two-sided Student’s *t* test). Data are presented as the mean ± SD of biological triplicates. The mean expression level of samples at 0 h was set to 1

To further investigate the effect of *EWS/ATF1* expression on OIS in sarcoma-iPSC-derived differentiated cells, we established mouse embryonic fibroblasts (MEFs) from sarcoma-iPSC chimeric embryos, which was followed by puromycin selection. We found that the sarcoma-iPSC MEFs harbor genetic aberrations, including a part of chromosomal abnormalities and mutations at *Plekhg5* and *Alk*, which are identical to those in the secondary sarcomas (Supplementary Fig. 4a). Notably, in sharp contrast to the active proliferation of *EWS/ATF1*-expressing sarcoma cells, *EWS/ATF1*-expressing sarcoma-iPSC MEFs ceased growth and changed morphology into a large and flat shape (Fig. 3a and Supplementary Fig. 4b–d). The senescence-associated β-galactosidase (SA β-gal)-positive cell ratio was significantly higher in *EWS/ATF1*-expressing MEFs than in non-expressing MEFs (Fig. 3b), suggesting that *EWS/ATF1* induces premature senescence in sarcoma-iPSC MEFs. Consistently, *EWS/ATF1* induced the expression of *Ink4a*, *Arf*, and *Cdkn1a* (*p21*) in sarcoma-iPSC MEFs (Fig. 3c). Moreover, knockdown of *Trp53* rescued the growth arrest phenotype (Fig. 3d and Supplementary Fig. 4e), which supports our conclusion that *EWS/ATF1* induces OIS in sarcoma-iPSC MEFs.

Conversely, SA β-gal-positive cells were increased by the withdrawal of *EWS/ATF1* in sarcoma cells (Fig. 3e). Furthermore, *Ink4a* and *Arf* expressions were rapidly increased after the withdrawal of *EWS/ATF1* in sarcoma cells (Fig. 3f), demonstrating that *EWS/ATF1* expression actively prevents senescence in sarcoma cells. Consistent with the opposing effects of *EWS/ATF1* expression on senescence, *Il-6*, a senescence-associated secretory phenotype-associated gene was inversely regulated in sarcoma cells and sarcoma-iPSC MEFs (Supplementary Fig. 4f). Collectively, our results indicate that sarcoma-iPSC mice develop secondary sarcomas in a cell-type-dependent manner that is associated with the opposing senescence response to *EWS/ATF1*. These results also suggest that cancer-related genetic abnormalities are not sufficient for senescence escape or eventual sarcoma development in most cell types in this model.

### Abnormal cell proliferation starts in peripheral nerves

**Fig. 4:**
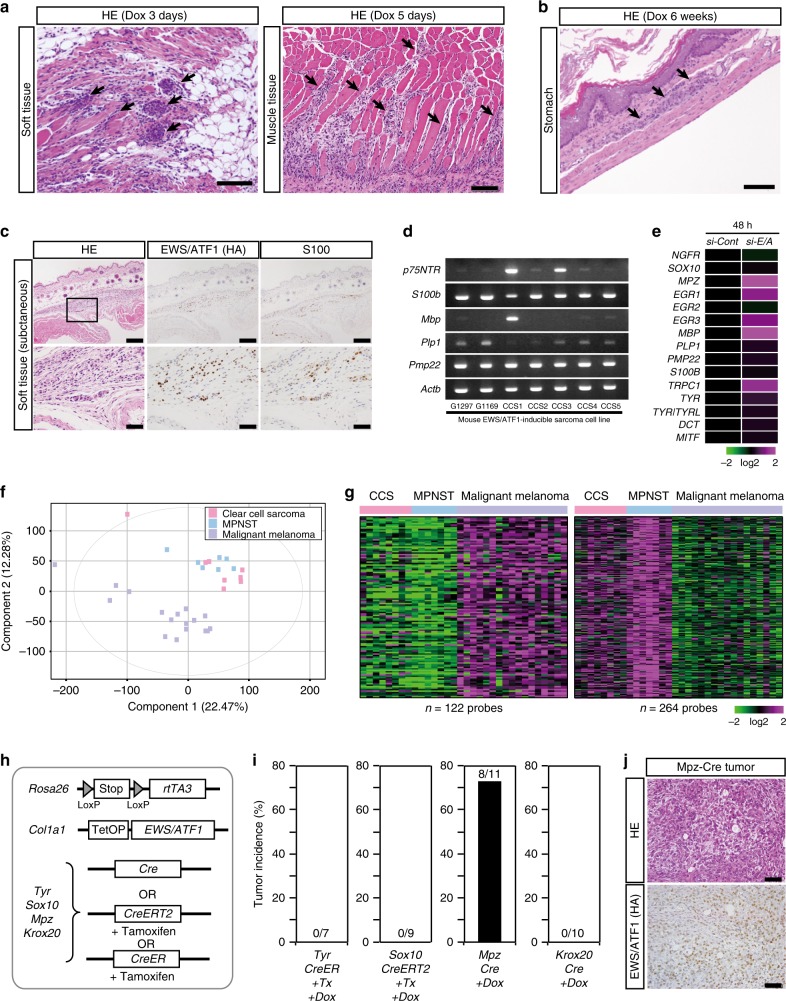
*EWS/ATF1* expression transforms peripheral nerve cells. **a** Hematoxylin and eosin (HE) staining of sarcoma-induced pluripotent stem cell (iPSC)-derived chimeric mice treated with doxycycline (Dox) for 3 days (left) or 5 days (right), respectively. Invasive growth of dysplastic cells is often observed along the course of peripheral nerves in the abdominal wall (arrows) and muscle tissue. Scale bars: 100 µm. **b** Secondary sarcoma development is found in Auerbach’s plexus at the gastric wall in sarcoma-iPSC-derived chimeric mice. Scale bar: 100 µm. **c** Microscopic sarcomas developed in the original clear-cell sarcoma (CCS) model mice after 6 weeks of Dox treatment. Microscopic tumor cells express S100. Scale bars: 200 µm (upper), 50 µm (lower). **d** Reverse transcription-polymerase chain reaction (RT-PCR) of murine *EWS/ATF1*-induced sarcoma cell lines demonstrates that most cell lines express Schwann cell-related genes. The number of PCR cycles was 35. **e** Heatmap of marker gene expression for neural crest derivatives at 48 h after knockdown of *EWS/ATF1* in a human CCS cell line (MP-CCS-SY) by microarray. **f** Principal component analysis (PCA) analysis of human CCSs, malignant peripheral nerve sheath tumors (MPNSTs), and malignant melanomas by microarray. Our microarray data included all 8 CCSs and 4 out of 7 MPNSTs along with published data of 3 MPNSTs (GSE60082) and all 17 melanomas (GSE30812)^50,51^. **g** Heatmap showing expression of genes that are up- or downregulated at least 3-fold in MPNSTs compared to malignant melanomas in CCSs. Color range is shown using a log 2 scale. **h** A schematic illustration of the genetic construct for Dox-inducible *EWS/ATF1* in neural crest derivatives. Mice with *Cre* allele were given Dox for 3 months starting at 4 weeks of age. Mice with *CreERT2* (or *CreER*) allele were given tamoxifen 3 times at 3 weeks of age, followed by Dox administration for 3 months starting at 4 weeks of age. **i** Tumor incidence after *EWS/ATF1* induction in neural crest derivatives for 3 months. Mice with *Mpz*-*Cre* allele developed sarcomas while other mice did not. **j** Histology and hemagglutinin (HA) staining of sarcoma tissue in mice with *Mpz*-*Cre* allele. The histology resembles human CCSs and *EWS/ATF1*-induced sarcomas in mice^9^. Scale bars: 50 µm

Taking advantage of the one-step and cell-type-specific nature of secondary sarcoma development in sarcoma-iPSC mice, we next attempted to identify a cell of origin for *EWS/ATF1*-induced sarcomas. Histological analysis of sarcoma-iPSC mice shortly after *EWS/ATF1* induction revealed that the abnormal cell growth often starts around the center of the abdominal wall (Fig. 1e and Supplementary Fig. 3c). Therefore, we next performed detailed sequential histological analysis of the abdominal wall after *EWS/ATF1* induction in sarcoma-iPSC mice together with ESC- and ETF-iPSC-derived mice. We noticed that invasive cancer cell growth often starts along the course of the peripheral nerves in the soft tissue and muscle tissue (Fig. 4a). Some early microscopic lesions resided inside the peripheral nerves and showed bundle-like patterns (Supplementary Fig. 5a, b). We also observed microscopic sarcoma development in the gastric wall, which mimics CCS of the gastrointestinal tract in humans (*n* = 1/23, Fig. 4b and Supplementary Fig. 5c)^17^, and in the mammary gland (*n* = 1/23). Consistent with the notion that *EWS/ATF1*-induced sarcomas arise from peripheral nerve cells, the early lesions express S100 protein (Fig. 4c and Supplementary Fig. 5a). We also found that *EWS/ATF1*-induced sarcoma cell lines express several Schwann cell marker genes such as *P75*^*NTR*^, *S100b*, *Mbp*, *Plp1*, and *Pmp22* in vitro (Fig. 4d). These results suggest that S100-expressing peripheral nerve cells contain a cell of origin for *EWS/ATF1*-induced sarcomas.

### Schwann cell-related expression profiles in human CCSs

It has been reported that human CCSs harbor shared properties with malignant melanomas^6^. Consistently, previous studies demonstrated a similarity in the global gene expression patterns of CCSs and malignant melanomas^18^. However, we observed that abnormal cell growth often starts along the course of peripheral nerves. Additionally, we found that *EWS/ATF1*-induced mouse tumors and human CCSs often express Schwann cell markers (Supplementary Fig. 5d). Together, these findings raised the possibility that peripheral nerve cells are a cell of origin for CCSs.

A previous study suggested that *EWS/ATF1*-induced sarcomas arise from neural crest derivatives^9^. Given that key driver oncogene expression represses the differentiation properties of cell of origin, we next examined the expression of marker genes of neural crest derivatives after *EWS/ATF1* knockdown in human CCSs (Fig. 4e, Supplementary Fig. 5e–g). *EWS/ATF1* knockdown led to a significant upregulation of melanocyte-related genes, such as *TYR*, *MITF*, and *DCT*. However, we also found that a majority of Schwann cell-related genes were similarly upregulated by *EWS/ATF1* knockdown in human CCS cells, suggesting that CCSs harbor transcriptional signatures of both melanocytes and Schwann cells.

Malignant peripheral nerve sheath tumor (MPNST) is a Schwann cell-derived sarcoma that preferentially occurs in neurofibromatosis type1 patients^19^. Of note, both clustering analysis and principal component analysis using microarray data revealed that most CCSs resemble more MPNSTs than malignant melanomas (Fig. 4f and Supplementary Fig. 5h). Moreover, the genes upregulated (*n* = 264 probe sets) and downregulated (*n* = 122 probe sets) in MPNSTs compared to malignant melanomas were similarly upregulated and downregulated in CCSs, respectively (Fig. 4g). Collectively, our results indicate that human CCSs exhibit shared properties with Schwann cells in the peripheral nerves and Schwann cell-derived sarcomas.

### Neural crest-derived nerve cells are a cell of origin

We next employed a genetic approach to determine a cell of origin for *EWS/ATF1*-induced sarcomas. For this purpose, *Cre* or *CreERT2* (or *CreER*) transgene driven by a cell-type-specific promoter was combined with *Rosa26*-*loxP-stop-loxP* (*LSL*)-*rtTA3* allele and *Col1a1:: tetO-EWS/ATF1* to induce *EWS/ATF1* upon Dox exposure in a cell-type-specific manner (Fig. 4h and Supplementary Fig. 5i). *Mpz-Cre* allele was used for *EWS/ATF1* induction in neural crest derivatives. Similarly, we used *Tyr-CreER* allele for the induction in melanocytes and *Sox10-CreERT2* allele (Supplementary Fig. 5j) for immature neural crest derivatives as well as melanocytes and Schwann cells. Additionally, *Krox20-Cre* allele was utilized to induce *EWS/ATF1* in Schwann cells. After treatment with Dox for 3 months, we found that mice containing *Mpz-Cre* allele developed CCS-like sarcomas, indicating that neural crest-derived cells are a cell of origin for *EWS/ATF1*-induced sarcomas (Fig. 4i, j). However, other *Cre* alleles failed to induce sarcomas (Fig. 4i), suggesting that cells expressing well-established markers for differentiated neural crest derivatives do not give rise to sarcomas after *EWS/ATF1* induction.

### *Tppp3* is a candidate marker for a cell of origin of the sarcoma

**Fig. 5:**
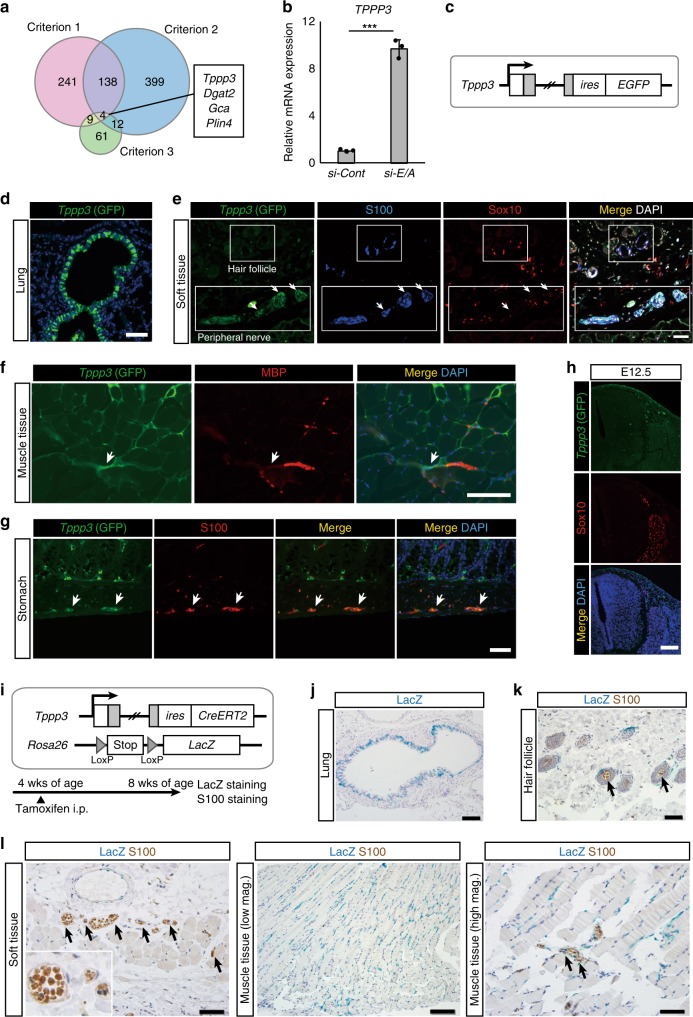
*Tppp3* as a candidate marker for cell of origin of clear-cell sarcomas. **a** Venn diagrams for the extraction of candidate genes of a cell of origin of *EWS/ATF1*-induced sarcoma. Criteria 1: mature peripheral nerve cells (4 months age)^20^ vs. immature peripheral nerve cells (5 days age)^20^, mature cells-high genes; fold change >4. Criteria 2: peripheral nerve cells^20^ vs. melanocytes^21^, mature peripheral nerve cells-high genes; fold change >10. Criteria 3: G1297 doxycycline (Dox) off vs. G1297 Dox on, G1297 Dox off-high genes; fold change >5. Overlapped four genes (*Tppp3*, *Dgat2*, *Gca*, and *Plin4*) are indicated. **b** A real-time quantitative RT-PCR (qRT-PCR) analysis of *TPPP3* after knockdown of *EWS/ATF1* in a human clear-cell sarcoma (CCS) cell line (MP-CCS-SY) (***P < 0.001, two-sided Student’s *t* test). Data are presented as the mean ± SD of biological triplicates. The mean expression level of *siControl*-treated cells was set to 1. **c** A schematic illustration of the genetic construct for visualization of *Tppp3* expression. **d** Immunofluorescence of *Tppp3-EGFP* homo mice (5 weeks age). Scale bar: 50 μm. **e** Green fluorescent protein (GFP) fluorescence (*Tppp3*) is also detectable in the peripheral nerve of soft tissue around the abdominal wall (arrows). Note that GFP expression is detectable in S100-positive cells in the peripheral nerve, while S100/Sox10-positive cells in the hair follicles are negative for GFP. Scale bar: 100 µm. **f** GFP (*Tppp3*) expression can be detectable at terminal fibers of peripheral nerves in the muscle tissue. Scale bar: 50 µm. **g** GFP (*Tppp3*) expression in the stomach. S100-positive cells in Auerbach’s plexus express GFP. Scale bar: 50 μm. **h** GFP (*Tppp3*) expression in Sox10-expressing migrating neural crest cells and dorsal root ganglion cells at E12.5. Scale bar: 100 µm. **i** A schematic illustration of the lineage tracing for *Tppp3*-expressing cells. Mice were treated with tamoxifen at 4 weeks old and sacrificed at 8 weeks old. **j** LacZ staining in bronchial epithelial cells. Scale bar: 100 µm. **k** Double staining for LacZ and S100 in the hair follicle. Scale bar: 50 µm. **l** LacZ-expressing cells are detectable inside peripheral nerves in the soft tissue (left) and muscle tissue (right) (arrows), which also express S100. LacZ staining is also observed in cells at the endomysium surrounding muscle fibers (middle). Scale bar: 50 µm (left and right), 100 µm (middle)

We therefore hypothesized that unclassified neural crest derivatives in peripheral nerves may be a cell of origin for *EWS/ATF1*-induced sarcomas. Accordingly, we next searched for candidate genes that are expressed in cell of origin for *EWS/ATF1*-induced sarcomas. For identification of the candidate genes, we used the following criteria: (1) genes that increase upon nerve cell differentiation, (2) genes that are expressed in nerve cells, but not in melanocytes. Given that cancer cells often recover the original cell properties by inhibition of key oncogenic signals, we also extract (3) genes that increase upon *EWS/ATF1* withdrawal in mouse CCS cells (G1297). According to published microarray data^20,21^, we extracted 393 genes, which were highly expressed in mature sciatic nerves (4 months of age) compared to immature sciatic nerve (5 days of age) (criterion 1) (cut-off fold change; 4-fold), and 553 genes that are highly expressed in mature sciatic nerves compared to melanocytes (criterion 2) (cut-off fold change; 10-fold). We also extracted 86 genes that increase after withdrawal of *EWS/ATF1* expression in sarcoma cells (G1297) (criterion 3) (cut-off fold change; 5-fold) (Fig. 5a and Supplementary Fig. 6a). Finally, four overlapping genes in three criteria were regarded as candidate marker genes for cell of origin for *EWS/ATF1*-induced sarcomas. Among four candidate genes, we focused on *Tppp3* since remarkable increment of *TPPP3* expression was observed by knockdown of *EWS/ATF1* in human CCS cell line (MP-CCS-SY) (Fig. 5b).

### *Tppp3* is expressed in peripheral nerve cells of adult mice

*Tppp3* is a tubulin polymerization-promoting protein family member 3 gene and is expressed in developing tendon sheath in the embryonic stage^22^. To determine the *Tppp3* expression pattern in adult tissues, we next established *Tppp3-EGFP* reporter knock-in mice (Fig. 5c and Supplementary Fig. 6b). *Tppp3* was expressed in bronchial epithelial cells of the lung (Fig. 5d) and squamous epithelium of the esophagus, which is consistent with the LacZ expression pattern in the KOMP expression atlas for *Tppp3* (https://www.kompphenotype.org/). Notably, *Tppp3* was also expressed in a subset of cells inside peripheral nerves of the subcutaneous tissues, which also expressed S100 (Fig. 5e and Supplementary Fig. 6c), but were not merged with Sox10 (Fig. 5e). S100/Sox10-positive melanocytes in hair follicles were negative for the reporter (Fig. 5e), affirming that *Tppp3* is highly expressed in peripheral nerve cells compared with melanocytes in adult mice. *Tppp3* expression was also observed at peripheral nerves in the muscle tissue (Fig. 5f) and the enteric nerve cells (Fig. 5g). Notably, Sox10-expressing migrating neural crest cells and dorsal root ganglion cells at E12.5 were not stained with enhanced green fluorescent protein (EGFP) (Fig. 5h), suggesting that *Tppp3* is not expressed in early derivatives of neural crest.

To further investigate the distribution of adult *Tppp3*-expressing cells in subcutaneous tissue, we established reporter mice harboring *Rosa26*-*loxP-stop-loxP* (*LSL*)-*LacZ*^23^ and *Tppp3-CreERT2* (*Rosa26*^*Stop-LacZ*^*/Tppp3*^*CreERT2*^) (Fig. 5i and Supplementary Fig. 6d). Four-week-old *Rosa26*^*Stop-LacZ*^*/Tppp3*^*CreERT2*^ mice were treated with tamoxifen and sacrificed 4 weeks later. Consistent with the results in *Tppp3-EGFP* reporter mice, bronchial epithelial cells of the lung exhibit LacZ staining (Fig. 5j), while S100-expressing hair follicle cells did not express LacZ (Fig. 5k). Notably, LacZ staining was detectable in a small subset of S100-expressing peripheral nerve cells in both soft tissue and muscle tissue, which corresponds with the location of the initial abnormal cell growth in sarcoma-iPSC mice (Fig. 4a). LacZ-positive cells were also observed in the perimysium that surrounds muscle fibers (Fig. 5l).

### *Tppp3-*expressing cells as a cell of origin of the sarcoma

**Fig. 6:**
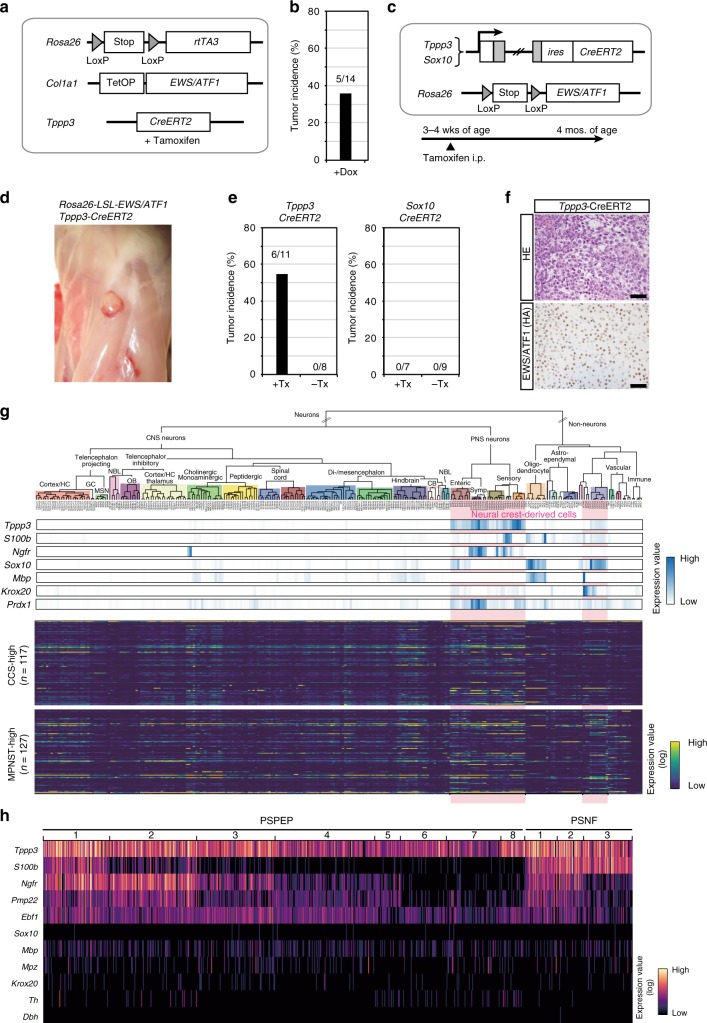
*Tppp3*-expressing cells, but not *Sox10*-expressing cells, give rise to sarcomas. **a** A schematic illustration of the genetic construct for doxycycline (Dox)-inducible *EWS/ATF1* in *Tppp3*-expressing cells. *CreERT2* driven by *Tppp3* promoter was used to transduce *EWS/ATF1*. Mice were given tamoxifen 3 times at 3 weeks of age, which was followed by Dox administration for 3 months starting at 4 weeks of age. **b** Five out of 14 mice developed tumors at 4 months of age. **c** A schematic illustration of the genetic construct for *EWS/ATF1* induction from *Rosa26* endogenous promoter in *Tppp3*- and *Sox10*-expressing cells (left, upper). Mice were given tamoxifen 3 times at 3 weeks of age and sacrificed after 3 months. **d** *Rosa26*^*stop-E/A*^*/Tppp3*^*CreERT2*^ mice developed macroscopic tumors around fascia. **e** Tumor incidence in mice with/without tamoxifen treatment is shown. Note that mice with *Tppp3-CreERT2* allele, but not *Sox10-CreERT2* allele developed tumors. **f** Histology of these mice recapitulates the results of other clear-cell sarcoma (CCS) model mice (upper panel). EWS/ATF1 expression in the tumor cells is detected by hemagglutinin (HA) immunostaining (lower panel). Scale bars: 50 µm. **g** Hierarchical taxonomic atlas of the mouse nervous system by single-cell RNA-sequencing (RNA-Seq) data^24^. Gene expression of neural crest derivative markers (upper panel) and CCS-/malignant peripheral nerve sheath tumor (MPNST)-overexpressed genes (lower panel) is shown along the cell-type taxonomy by heatmap. Neural crest-derived cell types are marked with a pink background. *Tppp3* expression is detectable in neural crest-derived clusters with neuron-like transcriptional signatures (upper panels). Note that most *Tppp3-*expressing clusters do not express *Sox10* or *Krox20*. **h** Single-cell expression data of neural crest-related genes. Heatmap of each row represents expression values from a single cell. A subset of *Tppp3*-expressing cells co-expresses *S100b*, *Ngfr*, *Pmp22*, and *Ebf1* at the single-cell level, which are often expressed in CCSs. Note that *Sox10* and *Krox20* are hardly expressed in a *Tppp3*-expressing cell. The PSPEP and PSNF nomenclatures of cell types refer to the classification shown in Zeisel et al.^24^ (mousebrain.org)

To examine whether *Tppp3*-expressing cells can give rise to *EWS/ATF1*-induced sarcomas, we established compound transgenic mice that harbor *Tppp3-CreERT2* allele together with *Rosa26*-*loxP-stop-loxP* (*LSL*)-*rtTA3* allele and *Col1a1:: tetO-EWS/ATF1*, in which *EWS/ATF1* can be induced with Dox exclusively in *Tppp3*-expressing cells after tamoxifen treatment (Fig. 6a). Notably, these mice developed CCS-like sarcomas after Dox treatment for 3 months (5/14) (Fig. 6b). To further confirm that *Tppp3*-expressing cells give rise to sarcomas, we also generated mice harboring *Tppp3-CreERT2* allele and *Rosa26*-*loxP-stop-loxP* (*LSL*)-*EWS/ATF1* allele (*Rosa26*^*Stop-E/A*^*/Tppp3*^*CreERT2*^) in which *EWS/ATF1* can be induced in *Tppp3*-expressing cells upon tamoxifen treatment (Fig. 6c and Supplementary Fig. 6e, f). We also generated transgenic mice containing *Sox10-CreERT2* alleles (*Rosa26*^*Stop-E/A*^*/Sox10*^*CreERT2*^), where *EWS/ATF1* can be induced in *Sox10*-expressing cells after tamoxifen injection (Fig. 6c and Supplementary Fig. 6f). Notably, CCS-like sarcomas were observed only in mice carrying *Rosa26*^*Stop-E/A*^*/Tppp3*^*CreERT2*^ at 3 months after tamoxifen treatment, while *Rosa26*^*Stop-E/A*^*/Sox10*^*CreERT2*^ mice never developed the sarcomas (Fig. 6d–f). Collectively, we concluded that *Tppp3*-expressing cells, but not *Sox10*-expressing cells, are a cell of origin for *EWS/ATF1*-induced sarcomas.

### Neural crest-derived cells express both *Tppp3* and *S100b*

We found that *Tppp3*-expressing cells can give rise to sarcomas and early sarcoma lesions often express S100, which suggests that *Tppp3*/*S100*-expressing neural crest-derived cells in peripheral nerves are a cell of origin for *EWS/ATF1*-induced sarcomas. Taking advantage of the taxonomic transcriptome atlas of the mouse nervous system^24^, we next investigated transcription profiles of *Tppp3*-expressing peripheral nerve cells. The hierarchical taxonomy based on a single-cell RNA-sequencing revealed the preferential expression of *Tppp3* at clusters of neural crest-derived cells in the nervous system (Fig. 6g). Notably, a subset of the *Tppp3*-expressing clusters expresses *S100b*. Furthermore, higher levels of *Tppp3* were observed in clusters with neuron-like transcription signatures, which do not express *Sox10* (Fig. 6g). Consistent with a previous study demonstrating that *EWS/ATF1* drives tumorigenesis in the *Prx1* lineage^10^, *Tppp3*-expressing clusters also expresses *Prdx1* (Fig. 6g).

To investigate transcriptional memory of a cell of origin, we next examined the expression pattern of uniquely overexpressed genes in human CCSs and MPNSTs using the taxonomic atlas of the mouse nervous system. Three representative neural crest-derived tumors, CCSs, MPNSTs, and malignant melanomas, were compared to extract uniquely overexpressed genes in CCSs (*n* = 117; cut-off fold change; 2-fold) and MPNSTs (*n* = 127; cut-off fold change; 2.5-fold). Notably, expression of MPNST-specific genes, but not CCS-specific genes, was significantly higher in *Sox10*/*Krox20*-expressing neural crest-derived cell clusters (Fig. 6g and Supplementary Fig. 6g). These results suggest that MPNSTs, but not CCSs, harbor transcriptional memory of Schwann cells in the peripheral nerves.

Finally, we examined a single-cell transcriptome of a *Tppp3*-expressing cell. We found that a subset of *Tppp3*-expressing cells co-expresses genes that are often highly expressed in CCSs at the single-cell level. A *Tppp3*-expressing cell simultaneously expresses *S100b*, *Ngfr*, *Pmp22*, and *Ebf1*, whereas *Sox10* and *Krox20* are hardly expressed in a *Tppp3*-expressing cell (Fig. 6h). Collectively, we propose that *Tppp3*-expressing and *Sox10*/*Krox20*-negative neural crest-derived peripheral nerve cells are a cell of origin for *EWS/ATF1*-induced sarcomas, at least in this transgenic mouse model.

### Cell-type-specific binding patterns of EWS/ATF1

**Fig. 7:**
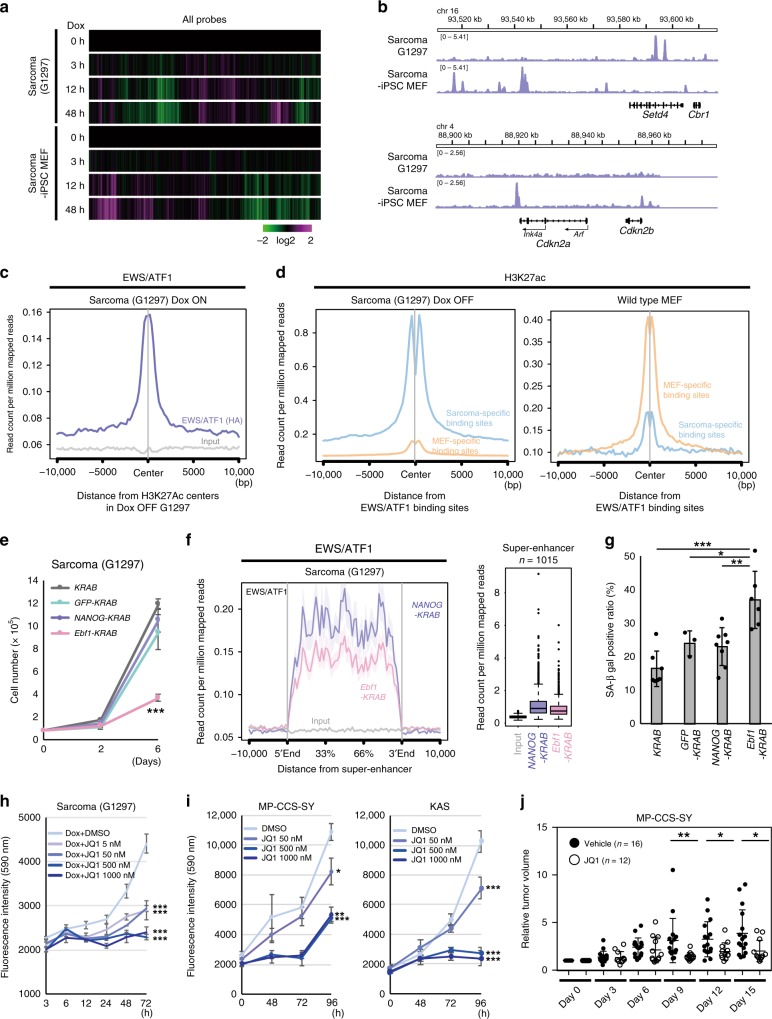
Cell-type-specific binding of EWS/ATF1 for sarcoma maintenance. **a** Microarray analysis of G1297 and sarcoma-induced pluripotent stem cell (iPSC)-derived mouse embryonic fibroblasts (MEFs) after exposure of doxycycline (Dox). Heatmap indicates that the two cell types respond differently to EWS/ATF1. Color range is shown using a log 2 scale. **b** Chromatin immunoprecipitation-sequencing (ChIP-Seq) data using hemagglutinin (HA) antibody demonstrate that EWS/ATF1 differentially binds to the genome of G1297 and sarcoma-iPSC MEFs. Representative genomic regions are shown. Note that EWS/ATF binds to *Cdkn2* locus only in sarcoma-iPSC MEFs. **c** ChIP-Seq analysis using HA antibody reveals that EWS/ATF1 binds to the H3K27ac-pre-marked region in Dox OFF G1297. **d** ChIP-Seq data for H3K27ac show that sarcoma-specific EWS/ATF1 binding sites possess more H3K27ac marks than MEF-specific EWS/ATF1 binding sites in G1297 (Dox OFF). Conversely, MEF-specific EWS/ATF1 binding sites possess more H3K27ac marks than sarcoma-specific EWS/ATF1 binding sites in wild-type MEFs (GSE31039). Blue line indicates H3K27ac enrichment in a sarcoma-specific EWS/ATF1 binding site, and orange line indicates H3K27ac enrichment in a MEF-specific EWS/ATF1 binding site. **e** Cell growth assay of G1297 introduced KRAB-fused transcription factors. *Ebf1-KRAB* transduction to G1297 causes a significant reduction of cell growth (***P < 0.001, two-sided Student’s *t* test). The mean ± SD of three independent biological experiments are shown, respectively. **f** *Ebf1-KRAB* transduction in G1297 for 48 h results in a significant reduction of EWS/ATF1 binding at a super-enhancer when compared with control *NANOG-KRAB*-inducing G1297 (*P* < 0.001, Mann–Whitney *U* test). **g** SA β-gal-positive ratio is increased by *Ebf1-KRAB* transduction for 4 days in G1297 (*P < 0.05, **P < 0.01, ***P < 0.001, two-sided Student’s *t* test). The mean ± SD of three to eight independent biological experiments are shown, respectively. **h** BET bromodomain inhibitor JQ1 inhibits the cell growth of G1297 (***P < 0.001, two-sided Student’s *t* test). The mean ± SD of three independent biological experiments are shown, respectively. **i** JQ1 inhibits the cell growth of human CCS cell lines (*P < 0.05, **P < 0.01, ***P < 0.001, two-sided Student’s *t* test). The mean ± SD of three independent biological experiments are shown, respectively. **j** JQ1 treatment suppresses human CCS cell growth in xenograft model (*P < 0.05, **P < 0.01, Mann–Whitney *U* test). Solid lines in each conditions indicate the mean and ± SD of each conditions (Vehicle; *n* = 16, JQ1; *n* = 12) are shown, respectively

We next considered the molecular mechanisms for cell type specificity of sarcoma development. Considering the heterogeneous nature of *Tppp3*-expressing cells, it seems technically difficult to isolate *Tppp3*-expressing cells that give rise to the sarcoma. Therefore, we compared sarcoma cells (G1297) and sarcoma-iPSC MEFs, two different cell types that share genetic abnormalities, which are associated with sarcoma development but exhibit opposing senescence responses and cellular kinetics after *EWS/ATF1* induction. We first examined the early transcriptional response after *EWS/ATF1* induction in the two cell types. Sarcoma cells (G1297, Dox OFF) and sarcoma-iPSC MEFs were harvested at 3, 12, and 48 h after Dox treatment and examined for global gene expressions. Notably, the early transcriptional response was considerably different between sarcoma cells and sarcoma-iPSC MEFs (Fig. 7a). Inversely regulated genes in the two cell types upon *EWS/ATF1* induction often contain cell cycle-associated genes, such as *Mki67*, *Ccna2*, and *Aurka* (data not shown), which is consistent with the opposing cellular kinetics after Dox treatment. Notably, we found a global downregulation of histone-coding genes in sarcoma-iPSC MEFs while they were generally upregulated in sarcoma cells upon *EWS/ATF1* induction (Supplementary Fig. 7a). Considering that decreased histone levels are implicated in senescence from yeast to mammals^25,26^, these findings further support our conclusion that *EWS/ATF1* induction leads to the opposing responses of senescence in the two different cell types.

Given that EWS/ATF1 affects gene expression via binding to cyclic AMP response element (CRE) of the genome using the DNA-binding domain of ATF1^8^, we compared the distribution of EWS/ATF1 binding in sarcoma cells (G1297) and sarcoma-iPSC MEFs at 12 h after Dox treatment by chromatin immunoprecipitation-sequencing (ChIP-Seq) analysis. Although there exists common EWS/ATF1 binding sites in the two cell types (*n* = 5109), we observed unique binding sites in G1297 and sarcoma-iPSC MEFs (*n* = 2981 and 15601, respectively) (Fig. 7b and Supplementary Fig. 7b, c). Notably, the increased binding of EWS/ATF1 was associated with increased expression in G1297 (Supplementary Fig. 7d). Indeed, consistent with the premature senescence phenotype and the increased expression of *Ink4a* and *Arf* in sarcoma-iPSC MEFs, EWS/ATF1 binding was detectable at *Cdkn2a* locus in sarcoma-iPSC MEFs but not in G1297 (Fig. 7b). Moreover, EWS/ATF1 was enriched at a super-enhancer in G1297 (Supplementary Fig. 7e)^27^. Together, these results suggest that the cell-type-specific binding of EWS/ATF1 is responsible for the cell-type-specific transcriptional profiling.

### H3K27ac-pre-marked regions are targets of EWS/ATF1 binding

We further asked why the EWS/ATF1-binding patterns were different in the two cell types. We focused on H3K27ac, an active epigenetic mark, in sarcoma cells (G1297) and MEFs before EWS/ATF1 induction. ChIP-Seq data for H3K27ac in both Dox OFF sarcoma cells (sarcoma cells without *EWS/ATF1* expression) and wild-type MEFs^28,29^ revealed that EWS/ATF1 was recruited to H3K27-pre-marked regions in both sarcoma cells and sarcoma-iPSC MEFs (Fig. 7c and Supplementary Fig. 7f, g). Furthermore, cell-type-specific binding sites were differentially pre-marked with H3K27ac in the two cell types: in Dox OFF sarcoma cells, sarcoma-specific binding sites were preferentially pre-marked with H3K27ac and less marked at MEF-specific binding sites (Fig. 7d); this relationship was conversed in wild-type MEFs (Fig. 7d). Collectively, these results suggest that EWS/ATF1 binds to genomic regions pre-marked with H3K27ac. However, H3K27ac-pre-marked sites did not always correspond to EWS/ATF1 binding sites (Supplementary Fig. 7b), which presumably reflects the different location of binding sites for the enhancer-related transcription factors (TFs) and ATF1.

### EWS/ATF binding in neural crest-related enhancers

An H3K27ac-marked region often corresponds to an enhancer. Given that TFs are involved in the establishment of enhancers, we next tried to identify TFs responsible for cell-type-specific EWS/ATF1 binding. HOMER (Hypergeometric Optimization of Motif EnRichment)-known motif analysis revealed a list of TF-binding motifs at sarcoma-specific and MEF-specific binding sites (Table 2). We found *Creb/Atf* and *Jun/Fos* motifs as the most enriched motifs in both sarcoma-specific and MEF-specific binding sites (Table 2), which is consistent with the fact that EWS/ATF1 binds to the genome through the DNA-binding domain of ATF1^8^. We also observed unique motifs at each binding site. Most notably, the unique enriched motifs at sarcoma-specific binding sites often included motifs of neural crest-related TFs, such as *Ebf1*, *Sox10*, and *Nur77* (*Nr4a1*), while those at MEF-specific binding sites included motifs of mesenchymal cell/connective tissue/embryonic tissue-related TFs, such as *Runx2*, *Tead4*, and *Meis1* (Table 2)^30–32^. These findings suggest that enhancers of cell of origin are targets of EWS/ATF1 bindings, which may account for the cell-type-specific patterns of EWS/ATF1 bindings.

**Table 2:** HOMER-known motif analysis

Sarcoma-specific binding sites							
Rank	Name	*P* value	# Target sequences with motif	% of Targets sequences with motif	# Background sequences with motif	% of Background sequences with motif	Motif
1	Atf1(bZIP)/K562-ATF1-ChIP-Seq(GSE31477)/Homer	1e-1014	2618	34.76%	2802.5	7.39%	Atf-related
2	Atf7(bZIP)/3T3L1-Atf7-ChIP-Seq(GSE56872)/Homer	1e-918	2141	28.43%	1983.2	5.23%	Atf-related
3	Atf2(bZIP)/3T3L1-Atf2-ChIP-Seq(GSE56872)/Homer	1e-849	1776	23.58%	1410.8	3.72%	Atf-related
4	c-Jun-CRE(bZIP)/K562-cJun-ChIP-Seq(GSE31477)/Homer	1e-734	1580	20.98%	1282.4	3.38%	Atf-related
5	CRE(bZIP)/Promoter/Homer	1e-610	1238	16.44%	912.8	2.41%	Atf-related
6	JunD(bZIP)/K562-JunD-ChIP-Seq/Homer	1e-584	902	11.98%	437.6	1.15%	Atf-related
7	Fra1(bZIP)/BT549-Fra1-ChIP-Seq(GSE46166)/Homer	1e-414	1582	21.00%	2270.4	5.99%	Atf-related
8	AP1(bZIP)/ThioMac-PU.1-ChIP-Seq(GSE21512)/Homer	1e-411	1882	24.99%	3136.4	8.27%	Atf-related
9	Atf3(bZIP)/GBM-ATF3-ChIP-Seq(GSE33912)/Homer	1e-406	1730	22.97%	2719.3	7.17%	Atf-related
10	BATF(bZIP)/Th17-BATF-ChIP-Seq(GSE39756)/Homer	1e-403	1726	22.92%	2720	7.17%	Atf-related
11	Fosl2(bZIP)/3T3L1-Fosl2-ChIP-Seq(GSE56872)/Homer	1e-352	1143	15.18%	1396	3.68%	Atf-related
12	Jun-AP1(bZIP)/K562-cJun-ChIP-Seq(GSE31477)/Homer	1.00E-287	881	11.70%	1007.1	2.66%	Atf-related
13	EBF(EBF)/proBcell-EBF-ChIP-Seq(GSE21978)/Homer	1.00E-126	412	5.47%	489.1	1.29%	Sarcoma-specific
14	Bach2(bZIP)/OCILy7-Bach2-ChIP-Seq(GSE44420)/Homer	1.00E-105	507	6.73%	823.9	2.17%	
15	EBF1(EBF)/Near-E2A-ChIP-Seq(GSE21512)/Homer	1.00E-100	1072	14.23%	2697.6	7.11%	Sarcoma-specific
16	Chop(bZIP)/MEF-Chop-ChIP-Seq(GSE35681)/Homer	1.00E-95	466	6.19%	762.9	2.01%	
17	Atf4(bZIP)/MEF-Atf4-ChIP-Seq(GSE35681)/Homer	1.00E-90	527	7.00%	970.4	2.56%	Atf-related
18	Nkx6.1(Homeobox)/Islet-Nkx6.1-ChIP-Seq(GSE40975)/Homer	1.00E-69	2961	39.31%	11288.6	29.77%	Sarcoma-specific
19	Lhx2(Homeobox)/HFSC-Lhx2-ChIP-Seq(GSE48068)/Homer	1.00E-64	1362	18.08%	4319.5	11.39%	Sarcoma-specific
20	Foxo1(Forkhead)/RAW-Foxo1-ChIP-Seq/Homer	1.00E-62	1985	26.35%	7022.5	18.52%	Sarcoma-specific
21	CEBP:AP1(bZIP)/ThioMac-CEBPb-ChIP-Seq(GSE21512)/Homer	1.00E-60	928	12.32%	2659	7.01%	Atf-related
22	FOXP1(Forkhead)/H9-FOXP1-ChIP-Seq(GSE31006)/Homer	1.00E-52	612	8.13%	1576.7	4.16%	Sarcoma-specific
23	NF1-halfsite(CTF)/LNCaP-NF1-ChIP-Seq(Unpublished)/Homer	1.00E-48	1655	21.97%	5889.6	15.53%	
24	MafA(bZIP)/Islet-MafA-ChIP-Seq(GSE30298)/Homer	1.00E-40	1027	13.64%	3381.4	8.92%	
25	FOXA1(Forkhead)/LNCAP-FOXA1-ChIP-Seq(GSE27824)/Homer	1.00E-38	1384	18.37%	4938.6	13.02%	Sarcoma-specific
26	Sox10(HMG)/SciaticNerve-Sox3-ChIP-Seq(GSE35132)/Homer	1.00E-34	1664	22.09%	6300.6	16.62%	Sarcoma-specific
27	Nur77(NR)/K562-NR4A1-ChIP-Seq(GSE31363)/Homer	1.00E-33	276	3.66%	607.9	1.60%	Sarcoma-specific
28	MITF(bHLH)/MastCells-MITF-ChIP-Seq(GSE48085)/Homer	1.00E-31	1187	15.76%	4275	11.27%	
29	Lhx3(Homeobox)/Neuron-Lhx3-ChIP-Seq(GSE31456)/Homer	1.00E-30	1778	23.61%	6933.2	18.29%	Sarcoma-specific
30	FOXA1(Forkhead)/MCF7-FOXA1-ChIP-Seq(GSE26831)/Homer	1.00E-30	1125	14.94%	4027.9	10.62%	Sarcoma-specific

MEF-specific binding sites							
Rank	Name	*P* value	# Target sequences with motif	% of Targets sequences with motif	# Background sequences with motif	% of Background sequences with motif	
1	Atf7(bZIP)/3T3L1-Atf7-ChIP-Seq(GSE56872)/Homer	1e-2516	6796	26.36%	1306.8	5.63%	Atf-related
2	Atf1(bZIP)/K562-ATF1-ChIP-Seq(GSE31477)/Homer	1e-2430	7999	31.03%	1895.2	8.16%	Atf-related
3	Atf2(bZIP)/3T3L1-Atf2-ChIP-Seq(GSE56872)/Homer	1e-2415	5702	22.12%	928.2	4.00%	Atf-related
4	c-Jun-CRE(bZIP)/K562-cJun-ChIP-Seq(GSE31477)/Homer	1e-2145	5184	20.11%	858.6	3.70%	Atf-related
5	JunD(bZIP)/K562-JunD-ChIP-Seq/Homer	1e-1716	2922	11.34%	294.1	1.27%	Atf-related
6	Atf3(bZIP)/GBM-ATF3-ChIP-Seq(GSE33912)/Homer	1e-1382	5991	23.24%	1705.1	7.34%	Atf-related
7	BATF(bZIP)/Th17-BATF-ChIP-Seq(GSE39756)/Homer	1e-1367	6005	23.30%	1726.5	7.43%	Atf-related
8	Fra1(bZIP)/BT549-Fra1-ChIP-Seq(GSE46166)/Homer	1e-1345	5400	20.95%	1438	6.19%	Atf-related
9	AP1(bZIP)/ThioMac-PU.1-ChIP-Seq(GSE21512)/Homer	1e-1309	6409	24.86%	1996.5	8.60%	Atf-related
10	CRE(bZIP)/Promoter/Homer	1e-1193	3207	12.44%	575.2	2.48%	Atf-related
11	Fosl2(bZIP)/3T3L1-Fosl2-ChIP-Seq(GSE56872)/Homer	1e-1052	3678	14.27%	853	3.67%	Atf-related
12	Jun-AP1(bZIP)/K562-cJun-ChIP-Seq(GSE31477)/Homer	1e-922	2881	11.18%	596.2	2.57%	Atf-related
13	Bach2(bZIP)/OCILy7-Bach2-ChIP-Seq(GSE44420)/Homer	1e-387	1701	6.60%	459.1	1.98%	
14	Chop(bZIP)/MEF-Chop-ChIP-Seq(GSE35681)/Homer	1.00E-297	1603	6.22%	497.1	2.14%	
15	Atf4(bZIP)/MEF-Atf4-ChIP-Seq(GSE35681)/Homer	1.00E-271	1769	6.86%	615.5	2.65%	Atf-related
16	CEBP:AP1(bZIP)/ThioMac-CEBPb-ChIP-Seq(GSE21512)/Homer	1.00E-189	3147	12.21%	1642.2	7.07%	Atf-related
17	RUNX-AML(Runt)/CD4 + -PolII-ChIP-Seq/Homer	1.00E-182	3251	12.61%	1732.1	7.46%	MEF-specific
18	RUNX(Runt)/HPC7-Runx1-ChIP-Seq(GSE22178)/Homer	1.00E-126	3229	12.53%	1891.4	8.14%	MEF-specific
19	NF-E2(bZIP)/K562-NF-E2-ChIP-Seq(GSE31477)/Homer	1.00E-111	507	1.97%	139.8	0.60%	MEF-specific
20	RUNX1(Runt)/Jurkat-RUNX1-ChIP-Seq(GSE29180)/Homer	1.00E-109	4110	15.94%	2629	11.32%	MEF-specific
21	TEAD(TEA)/Fibroblast-PU.1-ChIP-Seq(Unpublished)/Homer	1.00E-108	2673	10.37%	1546.3	6.66%	MEF-specific
22	RUNX2(Runt)/PCa-RUNX2-ChIP-Seq(GSE33889)/Homer	1.00E-106	3823	14.83%	2419.9	10.42%	MEF-specific
23	Bach1(bZIP)/K562-Bach1-ChIP-Seq(GSE31477)/Homer	1.00E-103	477	1.85%	132.4	0.57%	MEF-specific
24	Nrf2(bZIP)/Lymphoblast-Nrf2-ChIP-Seq(GSE37589)/Homer	1.00E-97	452	1.75%	126.4	0.54%	MEF-specific
25	MafA(bZIP)/Islet-MafA-ChIP-Seq(GSE30298)/Homer	1.00E-93	3410	13.23%	2158.3	9.29%	
26	TEAD4(TEA)/Tropoblast-Tead4-ChIP-Seq(GSE37350)/Homer	1.00E-88	3115	12.08%	1954.8	8.42%	MEF-specific
27	Meis1(Homeobox)/MastCells-Meis1-ChIP-Seq(GSE48085)/Homer	1.00E-84	6677	25.90%	4835.6	20.82%	MEF-specific
28	MITF(bHLH)/MastCells-MITF-ChIP-Seq(GSE48085)/Homer	1.00E-75	3891	15.09%	2623.1	11.30%	
29	NF1(CTF)/LNCAP-NF1-ChIP-Seq(Unpublished)/Homer	1.00E-65	1052	4.08%	536.5	2.31%	
30	Tbx20(T-box)/Heart-Tbx20-ChIP-Seq(GSE29636)/Homer	1.00E-64	1069	4.15%	549.7	2.37%	MEF-specific

Motifs appearing in sarcoma-specific and MEF-specific EWS/ATF1 binding sites are shown

*MEF* mouse embryonic fibroblasts, *ChIP-seq* chromatin immunoprecipitation-sequencing, *CRE* cyclic AMP response element, HOMER (Hypergeometric Optimization of Motif EnRichment)

### Epigenetic silencing induces growth arrest in sarcoma cells

Our cell-type-specific sarcoma model demonstrated that cancer genome requires specific cellular context to exert cancer properties, and suggested that enhancers in a cell of origin affect the genomic binding sites of key oncogenic proteins. Finally, we tried to alter the cancer cell fate by modulating the cell-type-specific binding pattern of EWS/ATF1. The Krüppel-associated box (KRAB) is a transcriptional repressor domain that interacts with histone deacetylase and induces epigenetic silencing^33^. Therefore, we attempted to induce silencing at sarcoma-specific EWS/ATF1 binding sites in sarcoma cells by overexpressing KRAB-fused Ebf1, since the *Ebf1* motif is the most abundant motif among sarcoma-specific motifs for EWS/ATF1 binding (Table 2). Notably, the introduction of the *Ebf1-KRAB* fusion gene resulted in remarkable growth inhibition of sarcoma cells, whereas the transduction of control *KRAB* fusion genes did not inhibit the growth of sarcoma cells (Fig. 7e and Supplementary Fig. 8a). Importantly, knockdown of *Ebf1* did not affect sarcoma cell growth (Supplementary Fig. 8b), suggesting that enhancer activity at *Ebf1* binding sites, but not *Ebf1* expression itself, has an impact on the growth of sarcoma cells. Consistent with this, human CCS cell line MP-CCS-SY exhibited growth inhibition upon the transduction of *KRAB* genes fused with *SOX10* and *BRN2*, both of which are important TFs for neural crest cell differentiation^34,35^ (Supplementary Fig. 8c).

Mechanistically, we found that *Ebf1-KRAB*-expressing sarcoma cells exhibit a reduced enrichment of EWS/ATF1 at the super-enhancer when compared with the control *NANOG-KRAB*-expressing sarcoma cells (Fig. 7f). Moreover, consistent with our finding that EWS/ATF1 actively prevents premature senescence in sarcoma cells, SA β-gal-positive cells were increased in *Ebf1-KRAB*-expressing sarcoma cells (Fig. 7g). Consistently, *Cdkn1a* and *Ink4a*, as well as *Il-6*, were upregulated in *Ebf1-KRAB*-expressing sarcoma cells when compared with control *NANOG-KRAB*-expressing sarcoma cells (Supplementary Fig. 8d).

Finally, we tested the effect of pharmacologic inhibition of the super-enhancer in CCS cells using BET bromodomain inhibitor JQ1. Notably, both mouse and human CCS cells showed reduced cell growth by JQ1 treatment in vitro (Fig. 7h, i), whereas the effect was not obvious in Ewing sarcomas that harbor *EWS/FLI1* fusion oncogene (Supplementary Fig. 8e, f). The inhibitory effect of JQ1 treatment on CCS cell growth was confirmed in a xenograft model of human CCS cells (Fig. 7j). Altogether, these results demonstrate that enhancers of cell of origin play an important role in both the development and maintenance of CCS, suggesting that they could make a promising therapeutic target.

## Discussion

Here we showed that sarcoma-iPSC mice exhibit extremely short latency for secondary sarcoma development after induction of the key driver oncogene. The fact that invasive growth starts as early as 5 days in a cellular context-dependent manner indicates that somatic cells with particular epigenetic regulation immediately turn into cancer cells in this model. Considering that most in vivo solid cancer models require considerable latency for cancer development, this model should provide a unique tool for studying many aspects of cancer biology. Indeed, taking advantage of the model, we here propose that *Tppp3*-expressing neural crest-derived cells are a cell of origin for mouse CCS cells and epigenetic regulation at the cell of origin affects the binding patterns of a key driver oncogenic protein, which leads to the establishment of a sarcoma-specific super-enhancer. Our results suggest that the epigenetic regulation of cell of origin may primarily play a critical role in the development and maintenance of cancer cells.

Genetic mutation of either *p53* or *Ink4a/Arf* locus bypasses the premature senescence induced by oncogenic *Ras*, demonstrating that genetic aberration is causative of the escape from senescence. Notably, we here show that sarcoma-iPSC mice immediately develop secondary sarcomas from peripheral nerve cells, but exhibit premature senescence in other tissues upon the induction of *EWS/ATF1*. Although the mechanism by which *Tppp3*-expressing cells can escape from senescence remains unclear, our results suggest that the escape from premature senescence is mediated by the cellular context with distinct enhancer landscapes, which highlights the impact of cell-type-related epigenetic regulation in cancer development. Consistent with this, we demonstrated that epigenetic silencing at cell-type-specific enhancers promotes premature senescence and induces a growth arrest phenotype in sarcoma cells. We propose that epigenetic regulations of the cell of origin could be targets for modulating cancer cell fate. Considering that key oncogenic signals often use TFs as effectors of the signal transduction, our findings might provide a general basis for cell type specificity in cancer development.

It is noteworthy that *EWS/ATF1*induced sarcomas from *Tppp3*-expressing neural crest-derived cells in peripheral nerves. A previous proteome analysis demonstrated that *Tppp3* messenger RNA (mRNA) and protein expression levels increase upon peripheral nerve cell differentiation, suggesting that more mature peripheral nerve cells express *Tppp3*^20,36^. Conversely, in the present study, *EWS/ATF1* expression failed to transform *Sox10*- and *Knox20*-expressing Schwann cells, which suggests that *Tppp3*-expressing cells may be a specialized differentiated cell type of neural crest-derived peripheral nerve cells. Notably, a single-cell transcriptome of a *Tppp3*-expressing cell revealed that a subset of a *Tppp3*-expressing cells simultaneously expresses *S100b*, *Ngfr*, *Pmp22*, and *Ebf1*, which are often highly expressed in CCSs, whereas *Sox10* and *Krox20* are hardly expressed in the *Tppp3*-expressing cell. Taken together, we propose that *Tppp3*-expressing and *Sox10*-negative neural crest-derived peripheral nerve cells are a cell of origin for *EWS/ATF1*-induced sarcomas. However, it should be noted that CCSs frequently express well-characterized markers of both Schwann cells and melanocytes, including *SOX10* and *TYR*. Notably, the taxonomic transcriptome atlas of the mouse nervous system suggested that oligodendrocytes express genes characteristic in neural crest-derived cells such as *Sox10*, which raised the possibility that reprogramming into neural crest-like state could occur even in non-neural crest derivatives^24^. It is possible that such reprogramming is associated with the expression of characteristic genes in a wide range of neural crest derivatives in CCSs^10^.

**Fig. 8:**
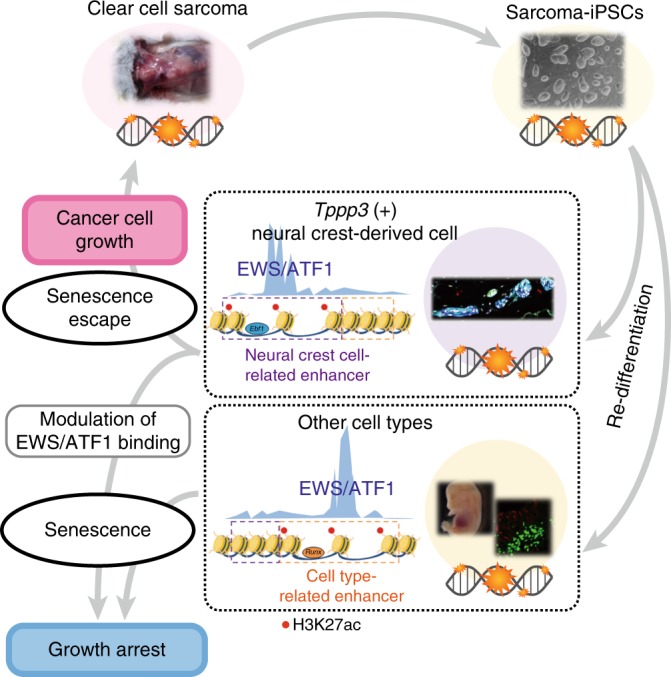
Schematic illustration of cell-type-specific sarcoma development. Induced pluripotent stem cells (iPSCs) from murine clear-cell sarcoma (CCS) cell lines that harbor sarcoma-associated genetic abnormalities develop secondary sarcomas in a cell-type-dependent manner, which is associated with a cell-type-specific response to premature senescence. Recruitment of EWS/ATF1 to enhancer regions is associated with the cell-type-specific response. Epigenetic silencing at these enhancers induces senescence and inhibits CCS cell growth through altered EWS/ATF1 binding

In summary, we established a cell-type-specific one-step in vivo sarcoma model using sarcoma-iPSCs. Using this unique cancer model, we determined *Tppp3*-expressing peripheral nerve cells as an origin of *EWS/ATF1*-induced sarcomas, provided insights into the cell type specificity for cancer development (Fig. 8).

## Methods

### Vector construction and gene targeting

To target *Rosa26-stop-EWS/ATF1* and *Rosa26-stop-rtTA3-ires-Venus* alleles, *EWS/ATF1-FLAG-HA* and *rtTA3-ires-Venus* cassettes were inserted into a targeting vector (pRosa26-DEST; obtained from Addgene)^37^. For the *Sox10-CreERT2*, *Tppp3-CreERT2*, and *Tppp3-EGFP* knock-in system, the Red/ET bacterial artificial chromosome (BAC) recombination system was used to introduce an *ires-CreERT2* or *EGFP-pA-rox-PGK-EM7-BsdR-pA-rox* cassette into 3′-UTR of *Sox10* BAC or *Tppp3* BAC, and targeting vectors with a DT-A-negative selection cassette were generated from these BACs. The obtained vectors were electropolated to V6.5 ESCs. ESCs were cultured with ES media containing 350 µg/ml G418 (Nacalai) or 15 µg/ml blasticidin S (Bsd, Funakoshi). Drug-resistant colonies were picked up and expanded. Correctly targeted ES clones were confirmed by Southern blotting. To generate *Rosa-M2rtTA; Col1a1*::*tetO- EWS/ATF1-ires-mCherry* ESCs, the *EWS/ATF1*-*FLAG-HA-ires-mCherry-pA* sequence was inserted into pBS31, which was electropolated into KH2 ESCs^38^. The established ESCs were used to generate chimeric mice by blastocyst injection.

### Cell lines

Mouse CCS cell lines (G1297, K11) were established from EWS/ATF1-inducible mice^9^. MP-CCS-SY and KAS are human CCS cell lines carrying EWS/ATF1 type 1 and type 2, respectively. MP-CCS-SY was provided by H. Moritake (University of Miyazaki, Miyazaki, Japan)^39^ and KAS was provided by T. Nakamura (Cancer Institute, Japanese Foundation for Cancer Research, Tokyo, Japan). The mouse Ewing sarcoma cell line (SCOS) was established from EWS/FLI1-inducible mice^40^. A673 was obtained from ATCC. TC135 was provided by Dr. T.J. Triche^41^. Cells were regularly checked for mycoplasma contamination (e-Myco Mycoplasma PCR Detection Kit, Life Diagnostics).

### In vivo experiment

All animal experiments were approved by the CiRA Animal Experiment Committee and IMSUT Animal Experiment Committee, and the care of the animals was in accordance with institutional guidelines. *EWS/ATF1*-inducible mice were treated with 200 μg/ml Dox-containing water and 10 mg/ml sucrose starting at the age of 4–6 weeks. For the Cre-mediated recombination, intraperitoneal injection of tamoxifen (2–4 mg/day × 2–4 times) was performed for mice harboring the *CreERT2* (or *CreER*) system at 3–4 weeks of age.

For xenograft studies, a total of 3 × 10^6^ sarcoma-derived iPS cells were transplanted to the subcutaneous tissue of NOD/ShiJic-scid Jcl mice (CLEA Japan) or BALB/cSLC-nu/nu mice (Japan SLC) to induce teratomas. MP-CCS-SY xenografts were established by injecting MP-CCS-SY cells (5 × 10^6^) in 30% Matrigel (BD Biosciences) into the flank of 7-week-old female BALB/cSLC-nu/nu nude mice (Japan SLC). One month after injection, mice with measureable tumors were divided into cohorts to be treated with JQ1 at 50 mg kg^−1^ or vehicle (5:95 = dimethyl sulfoxide:10% 2-hydroxypropyl-β-cyclodextrin) once a day for 15 days. Tumor volume was measured and calculated every 3 days.

### iPSC derivation and maintenance

iPSC derivation was performed by utilizing retroviral vectors (pMX-*OCT3/4*, pMX-*SOX2*, pMX-*KLF4*, and pMX-*cMYC*, obtained from Addgene)^42^. After the transduction of the reprogramming factors, sarcoma cells and ear tip fibroblasts were cultured in ESC media supplemented with human recombinant leukemia inhibitory factor (LIF) (Wako), 2-mercaptoethanol (Invitrogen) and 50 µg/ml l-ascorbic acid (Sigma). The established iPSCs were maintained with ESC media supplemented with LIF, 1 μM PD0325901 (Stemgent), and 3 μM CHIR99021 (Stemgent).

### Establishment of sarcoma-iPSC-derived MEFs

After blastocyst injection of sarcoma-derived iPSCs, chimeric embryos were harvested at E13.5, and MEFs were derived from the eviscerated and decapitated embryos with 0.25% trypsin. Because the *PGK-Puro-pA* selection cassette was placed at the *Rosa26*^*M2rtTA*^ knock-in allele^38^, MEFs were treated with 1 µg/ml puromycin (Sigma) for 1 week, and puromycin-resistant MEFs were used for further experiments.

### RT-PCR and real-time quantitative RT-PCR

RNA was extracted by using RNeasy Plus Mini Kit (Qiagen). Up to 1 µg RNA was used for the reverse transcription (RT) reaction into complementary DNA (cDNA). RT-polymerase chain reaction (RT-PCR) and real-time quantitative PCR were performed using Go-Taq Green Master Mix and Go-Taq qPCR Master Mix (Promega), respectively. Transcript levels were normalized by *β-actin* (mouse) and *GAPDH* (human). Ethical approval for the use of human tumor samples was granted by the ethics committee of Kyoto University Graduate School and Faculty of Medicine. All patients who donated tumor samples used in the study provided written, informed consent. PCR primers are listed in Supplementary Table 2.

### Bisulfite genomic sequencing

Bisulfite treatment was performed using EZ DNA Methylation-Gold Kit^TM^ (Zymo Research) according to the manufacturer’s protocol. Amplified products were cloned into pCR4-TOPO (Invitrogen) and transformed into DH5α. Colonies were randomly selected and sequenced with M13 forward and M13 reverse primers for each gene.

### Cell growth assay

Sarcoma cells and MEFs were plated into 12-well culture plates at a density of 8 × 10^4^ cells/well. The experiment was performed in triplicate, and each sample was measured twice. The number of cells was measured by automatic cell counter TC10^TM^ (Bio-Rad). For JQ1-treated cell growth assay, mouse and human clear-cell sarcoma cell lines (G1297, MP-CCS-SY, KAS) and mouse and human Ewing sarcoma cell lines (SCOS, TC135, A673) were plated into 96-well culture plates at density of 1 × 10^4^ cells/well. The experiment was performed in triplicate, and each sample was measured twice. The number of cells was measured by using alamarBlue cell viability reagent (Bio-Rad) according to the manufacturer’s protocol.

### Histological analysis and immunostaining

Tissues and tumor samples were fixed with 4% paraformaldehyde overnight and embedded in paraffin. Sections were stained with hematoxylin and eosin using standard protocol. For immunostaining, the antibodies used were anti-HA (C29F4) (Cell Signaling; dilution 1:100–500), anti-HA (Roche; dilution 1:50), anti-Ki67 (SP6) (Abcam; dilution 1:100–150), anti-BrdU (BU1/75 (ICR1)) (Abcam; dilution 1:100), anti-MBP (Abcam; dilution 1:100–200), anti-S100 (Dako; undiluted), anti-SOX10 (R&D; dilution 1:150), anti-GFP (4B10) (Cell signaling; dilution 1:150), anti-p53 (Ab240) (Abcam; dilution 1:250), and anti-p21 (HUGO291) (Abcam; dilution 1:100–500).

### ALP staining and SA β-gal staining

Cultured cells were washed with phosphate-buffered saline, fixed, and alkaline phosphatase (ALP) staining and SA β-gal staining were performed with ALP Staining Kit (Sigma-Aldrich) and Senescence β-Galactosidase Staining Kit (#9860S, Cell Signaling), respectively, according to the manufacturer’s protocol.

### siRNA transfection

Small interfering RNA (siRNA) transfection was performed using Lipofectamine RNAi Max (Invitrogen) at 70–80% confluency. The siRNA targeting the breakpoint of *EWS/ATF1* type 1 was used for the transfection^9^. Nontargeting siRNA was used as a control^41^.

### Transduction of KRAB-fused TFs

*KRAB*-fused genes (*Ebf1*, *SOX10*, *BRN2*, *NANOG*) and control gene (*GFP*) were inserted into pMXs retroviral vector. In all, 12.75 µg of these vectors and 5.625 µg of pCMV-VSV-G were transfected into 1 × 10^7^ competent cells using FuGENE HD (Roche) or PEI Max (PSI), and the viral supernatant was collected at 72 h after transfection. A total of 1 × 10^6^ of sarcoma cells were infected using the same amount of viral supernatant for 10 to 12 h.

### Array comparative genomic hybridization

Genomic DNA was extracted with PureLink^®^ Genomic DNA Mini Kit (Invitrogen). Array comparative genomic hybridization analysis was performed using SurePrint G3 Mouse Genome CGH Microarray Kit (Agilent) and analyzed using Agilent Genomic Workbench 7.0.

### Microarray analysis

Two hundred nanograms of total RNA prepared with RNeasy Mini Kit was subjected to cDNA synthesis with WT Expression Kit (Ambion), and the resultant cDNA was fragmented and hybridized to HuGene-1.0-st-v1 (Affymetrix) and Mouse Gene 1.0 ST Array (Affymetrix). After hybridization, GeneChip arrays were washed and stained by GeneChip Fluidics Station 450 (Affymetrix) and detected by Scanner 3000 TG system (Affymetrix) following the manufacturer’s standard protocols. The obtained data were analyzed using GeneSpring GX software (version 13.0, Agilent Technology).

### ChIP-sequencing

Anti-HA antibody (Nacalai, mouse monoclonal, HA124) and anti-acetyl histone H3 (Lys27) (Wako, mouse monoclonal, MABI0309) were used for the ChIP-Seq analysis. Purified ChIP DNA was used for the generation of sequencing libraries with TruSeq ChIP Sample Prep Kit (Illumina). The resultant DNA libraries were assessed on Agilent Bioanalyzer and quantified with KAPA Library Quantification Kits (Kapa Biosystems). The libraries were sequenced to generate single-end 109 bp reads using Illumina HiSeq. We analyzed ChIP-Seq data by mapping the reads using Bowtie2. The sequencing reads were aligned to mouse genome build mm9 or mm10. We used the MACS^43^ version 1.4.2 or 2.1.1 peak finding algorithm to identify regions of ChIP-Seq enrichment over the background with *P* value = 1 × 10^−9^. Super enhancers were identified by H3K27ac with ROSE pipelines^27,44^. To analyze and visualize the mapped reads, ngsplot was used^45^. The motif analysis was performed using HOMER software^46^.

### Exome analysis and direct sequencing

For the exome sequencing, 3 μg of the genome was extracted with PureLink Genomic DNA Kit. Whole-exome capture was performed with the SureSelect XT (Agilent Technologies). The exome libraries were then sequenced (109 bp paired-end reads) using a HiSeq2500 system (Illumina). The sequenced reads were mapped to the mouse reference genome (mm10) using Burrows–Wheeler Aligner (bwa-0.7.12)^47^ after trimming the adaptor sequences and low-quality bases by cutadapt-1.8.1^48^. Properly paired reads with a mapping quality of 30 or higher were extracted using samtools-1.2^49^, duplicate reads were marked using picard-tools-1.134 (https://broadinstitute.github.io/picard/), and local realignment was performed using Genome Analysis Tool Kit (GATK-3.4-0) for further analyses. Mutation calling was performed using scripts in the Genomon-exome pipeline (http://genomon.hgc.jp/exome/en/index.html). We selected somatic variants by removing the single-nucleotide polymorphisms (SNPs) and indels that were reported in dbSNP Build 142 and by removing the overlapping variants that were present in the V6.5, 129, B6, or DBA2 exome data (DRX077372, DRX077363, DRX077364). A list of the common mutations in G1297 and sarcoma-iPSC#3 is shown in Supplementary Table 1. For direct sequencing analysis, the PCR product containing the mutation candidate site was sequenced with the genetic analyzer ABI 3500xL (Applied Biosystems).

### Gene expression analysis in the mouse nervous system

Combinatorial gene expression patterns of neural crest-derived peripheral nervous system were analyzed by using the taxonomic transcriptome atlas of the mouse nervous system (http://www.mousebrain.org/).

### PCR primers

Primers used in this study are shown in Supplementary Table 2.

### Statistics

For comparison of the real-time quantitative RT-PCR data, Ki67-positive ratio, SA β-gal-positive ratio, and the cell growth assay, two-sided Student’s *t* test was used for the statistical analysis. Mann–Whitney *U* test was used for single-cell RNA-Seq analysis. Mann–Whitney *U* test was used for xenograft analysis. Differences were considered statistically significant at *P* < 0.05.

### Reporting summary

Further information on research design is available in the Nature Research Reporting Summary linked to this article.

## Supplementary information

Supplementary Information

Reporting Summary

## Data Availability

The ChIP-Seq data have been deposited in the Gene Expression Omnibus database under the accession code GSE124873. The microarray data have been deposited in the Gene Expression Omnibus database under the accession code GSE77203. All the other data supporting the findings of this study are available within the article and its Supplementary information files and from the corresponding author upon reasonable request. A reporting summary for this article is available as a Supplementary information file.
