# Supplementary material for: Cell-type dependent enhancer binding of the EWS/ATF1 fusion gene in clear cell sarcomas
Source: Nat Commun. 2019 Sep 5;10:3999. doi: 10.1038/s41467-019-11745-1 (PMC6728361; doi:10.1038/s41467-019-11745-1)
Supplement: Supplementary file 1 — Supplementary Information [file 41467_2019_11745_MOESM1_ESM.pdf]

Supplementary Information

Cell-type dependent enhancer binding of the EWS/ATF1 fusion gene in clear cell sarcomas

Komura et al.

Supplementary Figure 1

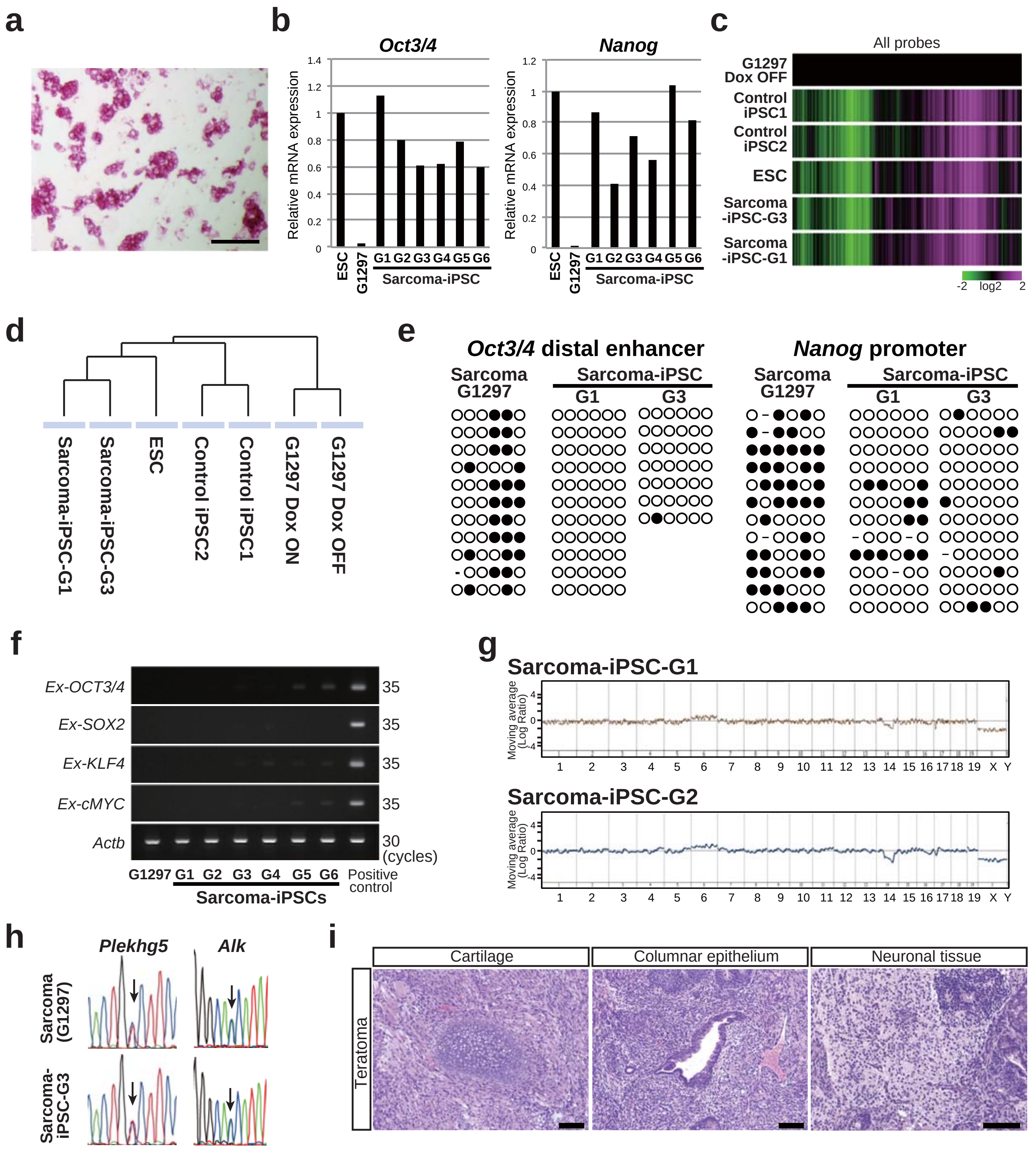

Supplementary Figure 1.
Derivation of sarcoma-iPSCs

a. Sarcoma-iPSCs exhibiting alkaline phosphatase activity. Scale bar: 300 μm.
b. qRT-PCR reveals that the expression levels of pluripotency-related genes (*Oct3/4* and *Nanog*) in established iPSC-like cell lines are equivalent to those in ESCs. Data are presented as the mean of technical triplicates. The expression level of ESCs was set to 1.
c. Microarray analysis of G1297 (Dox OFF), sarcoma-iPSCs and control iPSCs/ESCs (GSE45916) reveals that sarcoma-iPSCs acquired ESC- and normal iPSC-like gene expression signatures. Color range is shown using a log2 scale.
d. Hierarchical clustering analysis of G1297, sarcoma-iPSC, and control ES/iPSCs (GSE45916) by microarray. Global gene expression profiles of sarcoma-iPSCs are similar to those of ESCs.
e. Bisulfite sequencing analyses reveal that *Oct3/4* distal enhancer and *Nanog* promoter region are demethylated in sarcoma-derived iPSC-like cells. White and black circles indicate non-methylated and methylated cytosine at CpG sites, respectively.
f. RT-PCR shows the silencing of the 4 exogenous factors in established sarcoma-iPSCs lines. The number of PCR cycles is also indicated.
g. Shared chromosomal aberrations with G1297 are detected in sarcoma-iPSC lines G1 and G2.
h. Direct sequencing confirms shared missense mutations of the *Plekhg5* and *Alk* genes in G1297 and sarcoma-derived iPSC-like cells (clone G3).
i. Sarcoma-derived iPSC-like cells generated teratomas in the subcutaneous layer of immunocompromised mice. Scale bars: 100 μm.

Supplementary Figure 2

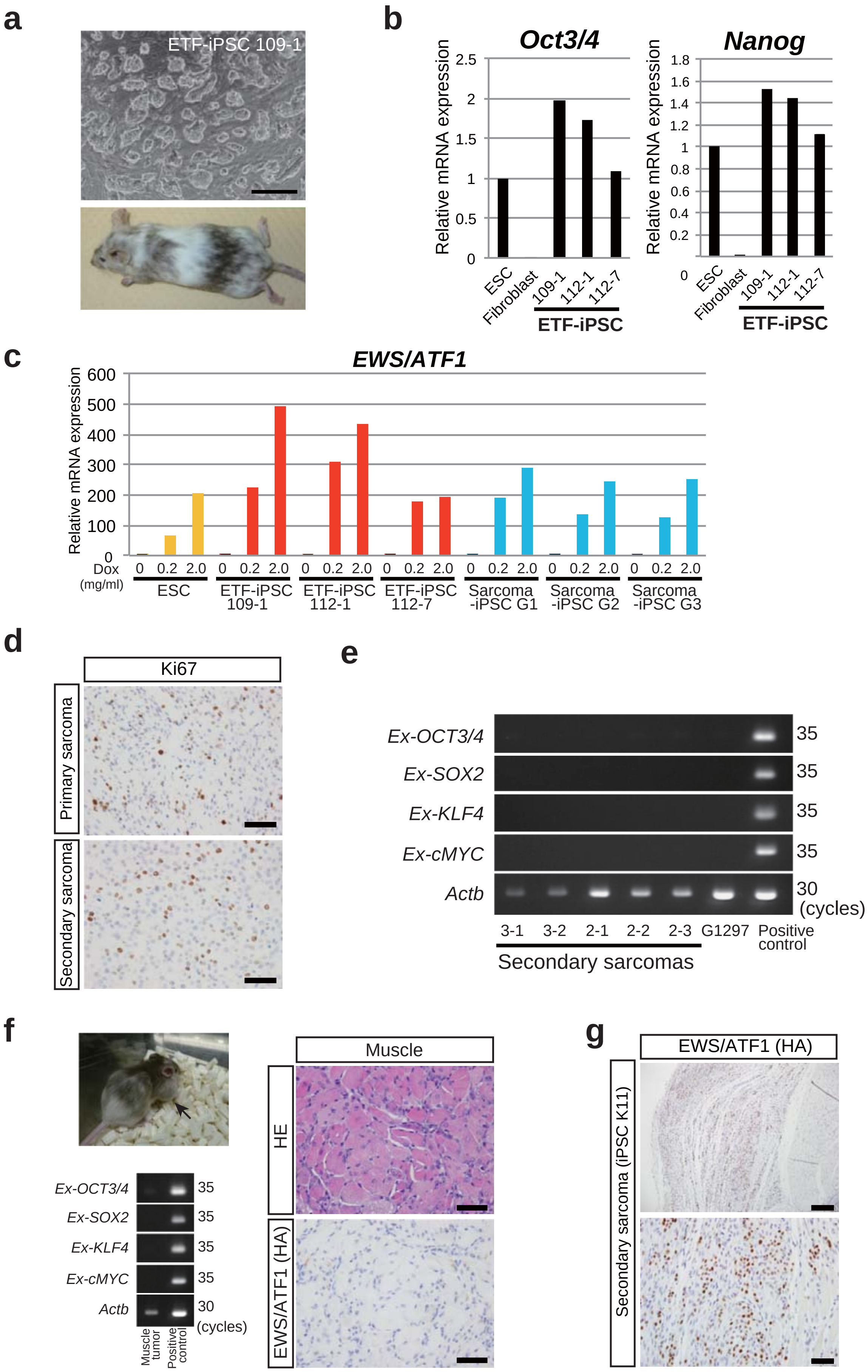

Supplementary Figure 2.

Sarcoma development in *EWS/ATF1*-inducible iPSC and ESC mice

a. ETF-iPSCs were established from ear tip fibroblasts of CCS model mice harboring a Dox-inducible *EWS/ATF1* allele with the equivalent reprogramming method used for sarcoma-iPSCs derivation. ETF-iPSCs contributed to somatic cells of the chimeric mice after injection into blastocyst. Scale bar: 300 µm.
b. qRT-PCR reveals that the expression levels of pluripotency related genes (*Oct3/4* and *Nanog*) in established ETF-iPSCs are equivalent to those in ESCs. Data are presented as the mean of technical triplicates. The expression level of ESCs was set to 1.
c. Comparison of *EWS/ATF1* expression level between ESCs, ETF-iPSCs, and sarcoma-iPSCs. Sarcoma-iPSCs exhibit similar expression levels of *EWS/ATF1* to control ESCs/iPSCs. Data are presented as the mean of technical triplicates. The expression level of ESC Dox0 was set to 1. Dox concentrations are 0, 0.2 and 2.0 µg/ml, respectively, and Dox exposure time is 8 hours.
d. Ki67 staining for primary and secondary sarcomas. Scale bars: 50 µm.
e. RT-PCR shows no obvious reactivation of exogenous 4 factors in secondary sarcomas. The number of PCR cycles is indicated.
f. Dox-independent rhabdomyosarcoma was observed in 1 of 7 sarcoma-iPSC mice without Dox treatment. No reactivation of reprogramming factors is detected. HE staining of rhabdomyosarcoma (top) and negative HA immunostaining (bottom) are shown. Scale bars: 50 µm.
g. HA (EWS/ATF1) staining in secondary sarcomas in K11-iPSC-derived chimeric mice. Scale bar: 200 µm (upper), 50 µm (lower).

Supplementary Figure 3

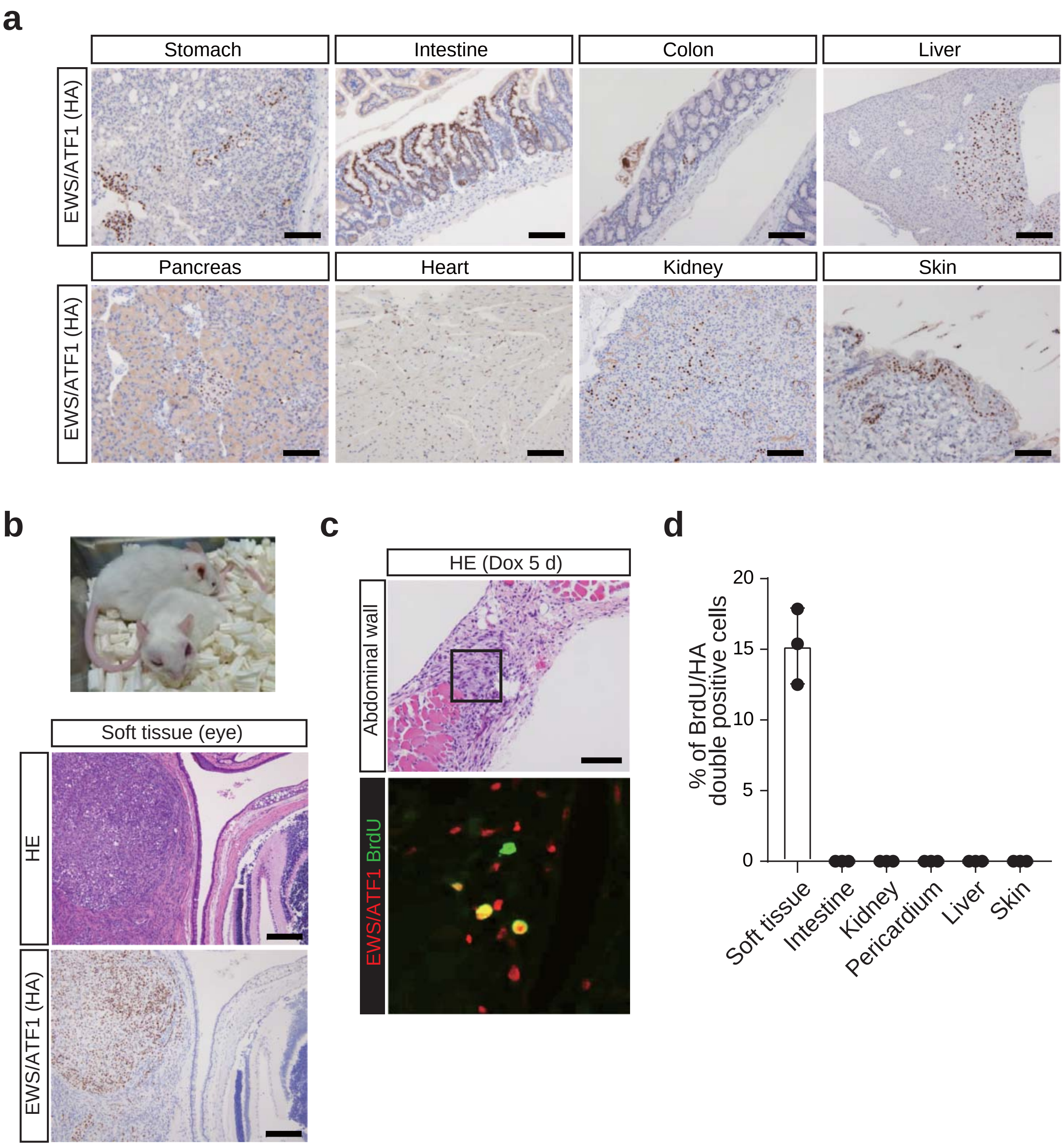

Supplementary Figure 3.

***EWS/ATF1* does not induce secondary sarcomas in a wide variety of cell types**

a. Immunohistochemistry for EWS/ATF1 using HA antibody. Representative images demonstrating that EWS/ATF1-expressing cells are detectable in various organs and tissues in sarcoma-iPSC chimeric mice after Dox treatment for 5-7 days. Scale bars: 100 μm.
b. Secondary sarcoma development was found in soft tissue outside of the eyes. Anti-HA antibody is used to detect EWS/ATF1. Scale bars: 100 μm.
c. Dysplastic cells were often observed in the center of the abdominal wall (upper panel). Scale bars: 100 μm. A lower panel shows immunofluorescence of the boxed region in the upper image using HA and BrdU antibodies. In sharp contrast to other organs, EWS/ATF1-expressing dysplastic cells at Day 5 display a BrdU signal, indicating escape from premature senescence.
d. Quantitative analysis for BrdU positive cells in HA positive cells in different organs. The mean ± SD of 3 independent biological experiments are shown, respectively.

Supplementary Figure 4

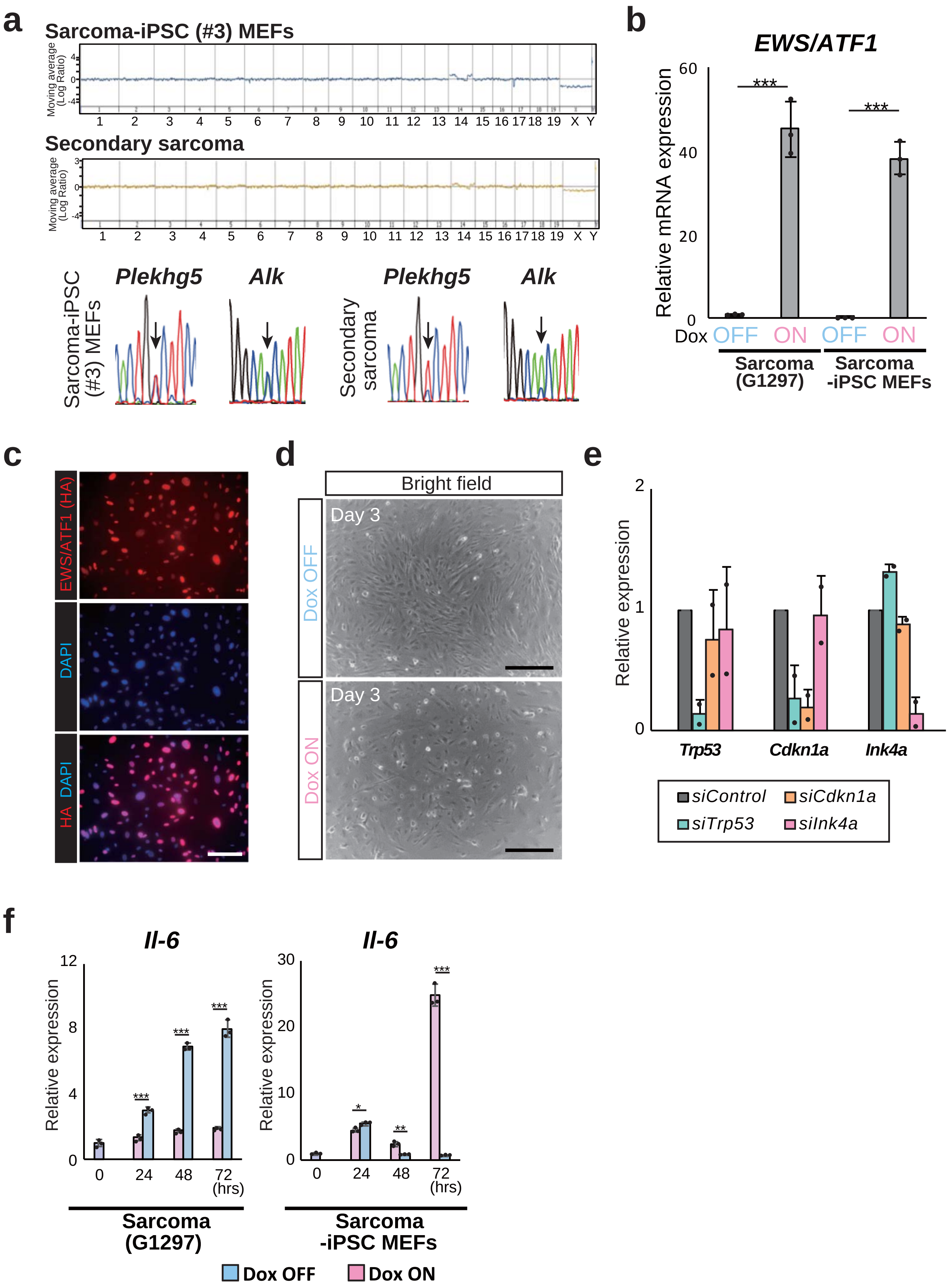

Supplementary Figure 4.
Sarcoma iPSC-derived MEFs exhibit growth arrest phenotype

a. Array CGH analysis reveals that sarcoma-iPSC MEFs and secondary sarcoma harbor some G1297-specific chromosomal abnormalities. Exome sequencing shows *Plekhg5* and *Alk* mutations in both sarcoma-iPSC MEFs and secondary sarcoma.
b. qRT-PCR analysis reveals comparative expression levels of *EWS/ATF1* in G1297 and sarcoma-iPSC MEFs (***P < 0.001, two-sided Student's t-test). Data are presented as the mean ± SD of triplicates. The mean expression level of G1297 Dox0 was set to 1. Dox concentration is 0.2 μg/ml, and Dox exposure time is 48 hours. The mean ± SD of 3 independent biological experiments are shown, respectively.
c. Immunocytochemistry of sarcoma-iPSC MEFs using HA antibody reveals that most MEFs expressed EWS/ATF1. Dox exposure time is 12 hours. Scale bar: 300 μm.
d. *EWS/ATF1* induction in sarcoma-iPSC MEFs results in morphological changes and growth inhibition. Dox exposure time is 3 days. Scale bars: 300 μm.
e. Knockdown efficiency for senescence-related genes. Data are presented as the mean ± SD of two independent experiments. The mean expression level of *siControl*-treated cells was set to 1.
f. A qRT-PCR analysis for *Il-6* in sarcoma cells and sarcoma-iPSC MEFs. Sarcoma cells exhibit an increased expression of *Il-6* upon *EWS/ATF1* withdrawal, while sarcoma-iPSC MEFs show an increased *Il-6* expression upon *EWS/ATF1* induction (*P < 0.05, **P < 0.01, ***P < 0.001, two-sided Student's t-test). Data are presented as the mean ± SD of independent biological triplicates. The mean expression level of samples at 0 h was set to 1.

Supplementary Figure 5

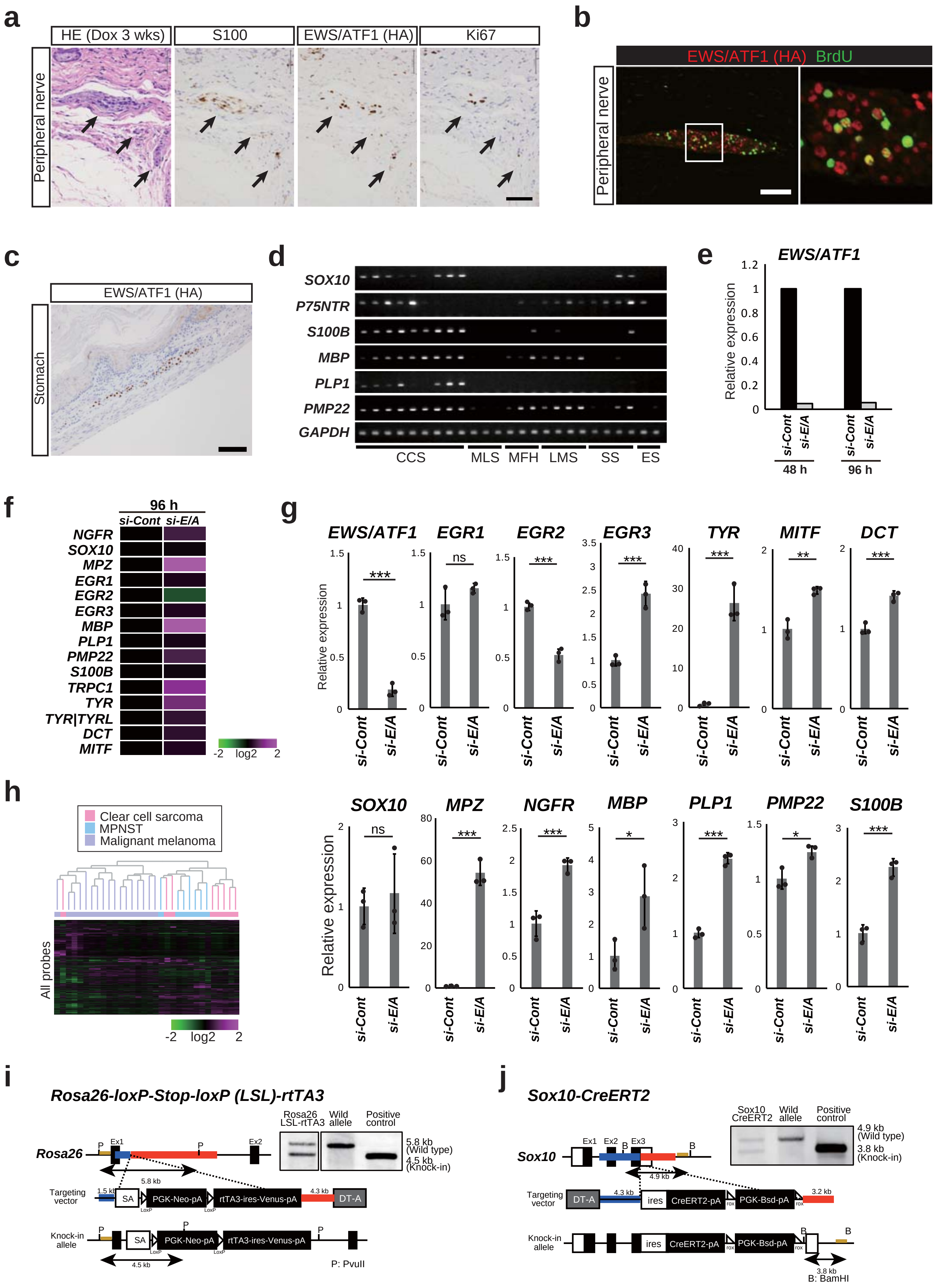

Supplementary Figure 5.
Murine and human CCSs harbor Schwann cell properties

a. Three weeks Dox treatment on a *EWS/ATF1*-inducible mouse resulted in the proliferation of peripheral nerve cells. Arrows indicate peripheral nerves. Scale bar: 50 μm.
b. EWS/ATF1-expressing microscopic tumor cells in ETF-iPSCs chimeric mice after 4 weeks of Dox treatment (left) merged with BrdU signals (right). Scale bar: 100 μm.
c. HA staining reveals EWS/ATF1 expression in microscopic sarcoma cells in Auerbach's plexus at gastric wall. Scale bar: 100 μm.
d. RT-PCR of human surgically-resected sarcoma samples. MLS, malignant liposarcoma; MFH, malignant fibrous histiocytoma; LMS, leiomyosarcoma; SS, synovial sarcoma; ES, Ewing sarcoma. Lanes 8, 9, and 24 are cell lines KAS, MP-CCS-SY, and TC135, respectively. The number of PCR cycles is 35.
e. Knockdown efficiency for *EWS/ATF1* in MP-CCS-SY samples used in microarray analysis. Data are presented as the mean of technical triplicates. The expression level of *siControl*-treated cells was set to 1.
f. Microarray analysis of marker genes for neural crest derivatives at 96 hours after knockdown of *EWS/ATF1* in MP-CCS-SY.
g. A qRT-PCR analysis of marker genes for neural crest derivatives after knockdown of *EWS/ATF1* in MP-CCS-SY. Data are presented as the mean ± SD of independent technical triplicates. The mean expression level of *siControl*-treated cells was set to 1. (ns; not significant, $*P < 0.05$, $**P < 0.01$, $***P < 0.001$, two-sided Student's t-test)
h. Hierarchical clustering analysis and heatmap of global gene expression in human CCSs, MPNSTs, and malignant melanomas by microarray. Color range in heatmap is shown using a log2 scale.
i. Schematic representation of the *Rosa26* targeting allele and Southern blot analysis of the obtained clone. Venus fluorescence is hardly detectable in the targeted cells.
j. Schematic representation of the *Sox10* targeting allele and Southern blot analysis of the obtained clone. ires, internal ribosomal entry site; Bsd, blasticidin resistant gene; PGK, PGK promoter; pA, poly(A) sequence; DT-A, diphtheria toxin A.

Supplementary Figure 6

a

Criterion 1

Gene symbol	Sciatic nerve Postnatal day 5	Sciatic nerve 4 months
Tppp3	777.4375	3340.4214
Gca	41.13325	185.52594
Gca	116.74856	438.3229
Plin4	166.25044	3350.2576
Dgat2	208.93199	889.275
Dgat2	236.84724	2019.1979

Criterion 2

Gene symbol	Melanocytes	Sciatic nerve
Tppp3	82.07335	3733.0137
Gca	17.619337	416.95166
Gca	10.918382	249.06717
Plin4	58.9481	3811.3484
Dgat2	107.58669	855.31305
Dgat2	135.11526	3053.8433

Criterion 3

Gene symbol	G1297 Dox ON	G1297 Dox OFF
Tppp3	623.8184	3406.1687
Gca	90.183334	571.5056
Plin4	127.26311	881.4622
Dgat2	207.6215	1791.6649

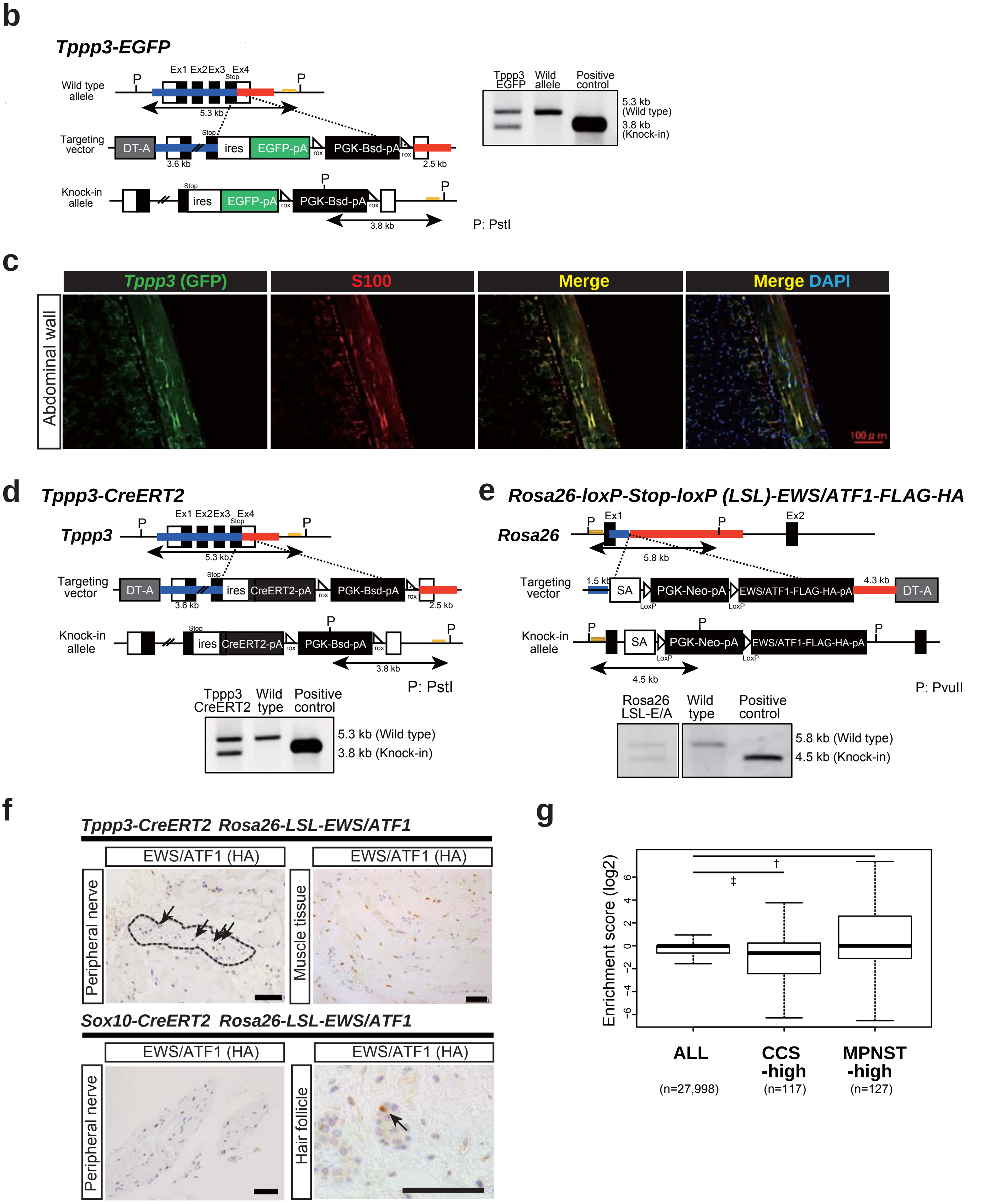

Supplementary Figure 6.
Visualization of *Tppp3* expression and conditional *EWS/ATF1* transduction system

a. The lists of microarray data regarding overlapped 4 genes. Normalized values are indicated.
b. Schematic representation of the *Tppp3* targeting allele. The *ires-GFP* sequence with a stop cassette is inserted into 3'UTR of the *Tppp3* locus. Southern blot analysis using a 3' external probe shows successful homologous recombination.
c. Immunofluorescence of the subcutaneous tissue of *Tppp3-EGFP* homo mouse (5 weeks old) using GFP and S100 antibodies. GFP fluorescence (green) is detectable in S100-positive peripheral nerve of soft tissue around the abdominal wall. Scale bar: 100 μm.
d. Schematic representation of the *Tppp3* targeting allele. The *ires-CreERT2* sequence with a stop cassette is inserted into 3'UTR of the *Tppp3* locus. Southern blot analysis of both obtained clones using a 3' external probe shows successful homologous recombination.
e. Schematic representation of the *Rosa26* targeting allele. The *EWS/ATF1-FLAG-HA* sequence with a stop cassette is inserted into intron 1 of the *Rosa26* locus. SA, splice acceptor; Neo, neomycin resistant gene. Southern blot analysis of the obtained clone using a 5'external probe shows successful homologous recombination.
f. $Rosa26^{stop\text{-}E/A}/Tppp3^{CreERT2}$ mice reveals EWS/ATF1 expression in the peripheral nerves at 1 week after tamoxifen injection (upper panel). $Rosa26^{stop\text{-}E/A}/Sox10^{CreERT2}$ mice show EWS/ATF1 expression in both peripheral nerves and hair follicles after tamoxifen injection. EWS/ATF1 expression is detected by HA-immunostaining (lower panel). Arrows indicate the nuclear expressions of EWS/ATF1. Scale bars: 50 μm.
g. Enrichment score [24] of CCS-/MPNST-overexpressed genes in *Sox10*/*Krox20*-expressing neural crest-derived cells with non-neuronal transcriptional signatures. Upregulated genes in MPNSTs are preferentially expressed in *Sox10*/*Krox20*-expressing cells (*Sox10* >1.0 or *Krox20* >0.1) while those in CCSs are not. Solid lines in each box indicate the median. The bottom and top of the boxes are lower and upper quartiles, respectively. Whiskers extend to ±1.5 interquartile range (IQR). (†, more enriched, $P<0.01$; ‡, less enriched, $P < 0.0001$, Mann-Whitney U-test)

Supplementary Figure 7

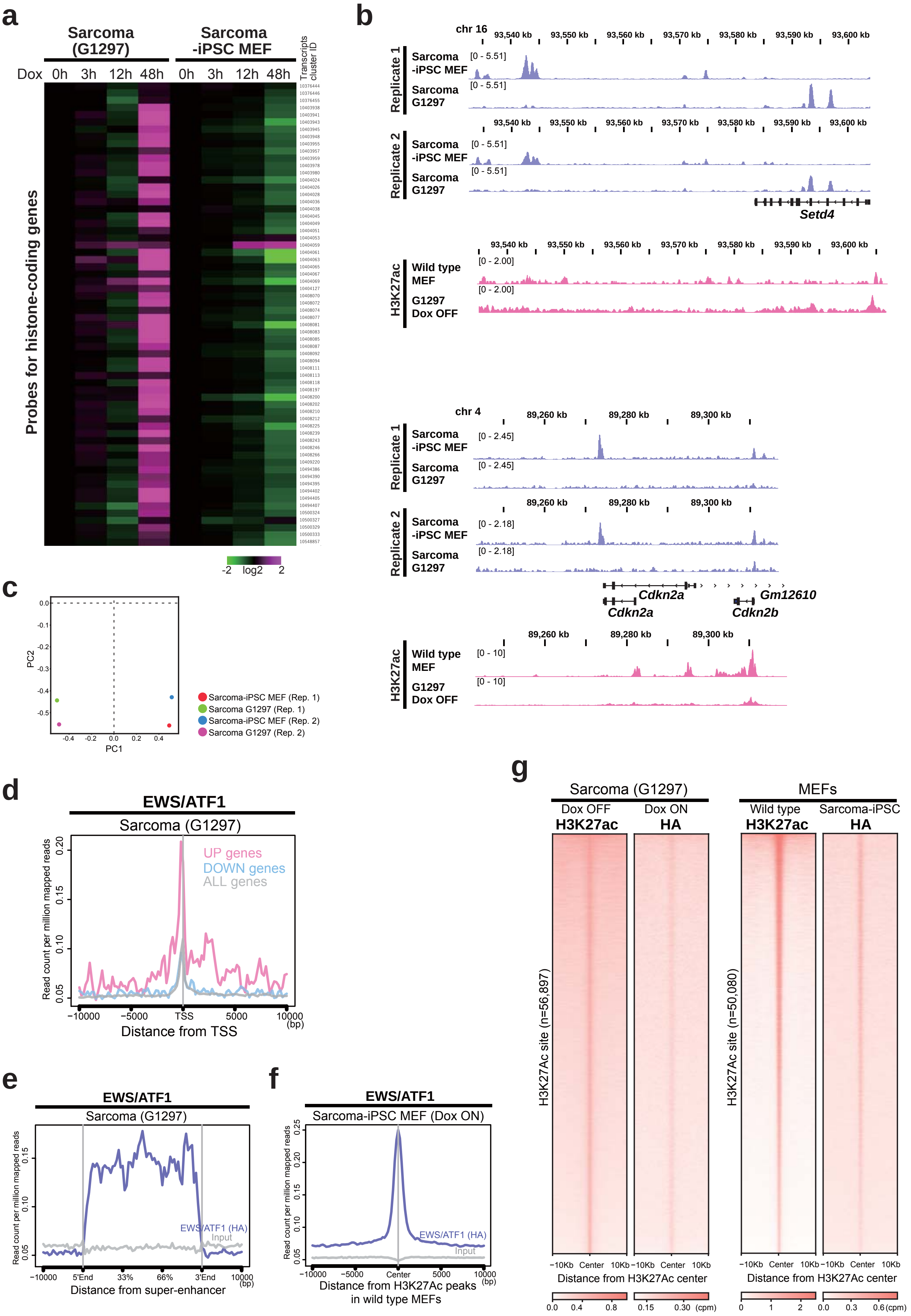

Supplementary Figure 7.

Cell type-specific recruitment of EWS/ATF1 plays a role in sarcoma cell growth

a. Heatmap of histone-coding gene expression in G1297 and sarcoma-iPSC derived MEFs after exposure of Dox. Note that the two cell types respond inversely to EWS/ATF1. Color range is shown using a log2 scale. Transcripts cluster IDs in Affymetrix Mouse Gene 1.0 ST Array are shown in the right column.

b. ChIP-seq analysis using HA antibody exhibits the similar EWS/ATF1 binding patterns in two biological replicates. However, H3K27ac pre-marked sites do not correspond to EWS/ATF1 binding sites in these regions, suggesting that H3K27ac pre-marked sites are not always targets of EWS/ATF1 binding, which presumably reflects the different location of binding sites for the enhancer-related transcription factors and ATF1.

c. PCA analysis with ChIP-seq datasets using HA antibody in G1297 and sarcoma-iPSC MEFs. The global enrichment pattern in each cell type is similar in two independent biological replicates.

d. ChIP-seq data of G1297 using HA antibody. An enrichment of EWS/ATF1 is observed around the TSSs of upregulated genes after *EWS/ATF1* induction, but not at downregulated genes.

e. ChIP-seq analysis reveals that EWS/ATF1 is enriched at a super-enhancer of sarcoma cells (G1297), suggesting that EWS/ATF1 plays a role in establishing the super-enhancer in sarcoma cells.

f. ChIP-seq analysis of sarcoma-iPSC MEFs shows that EWS/ATF1 binding enriches at the H3K27ac pre-marked and open chromatin region in wild type MEFs.

g. Heatmap of ChIP-seq intensities (copy per million, cpm). Normalized intensities for H3K27ac and HA (HA-tagged EWS/ATF1) within 10 kb +/-from the center of H3K27ac-marked site are shown. Heatmaps were ranked according to H3K27ac enrichment. EWS/ATF1 is enriched at most H3K27Ac pre-marked regions in both sarcoma cells and MEFs.

Supplementary Figure 8

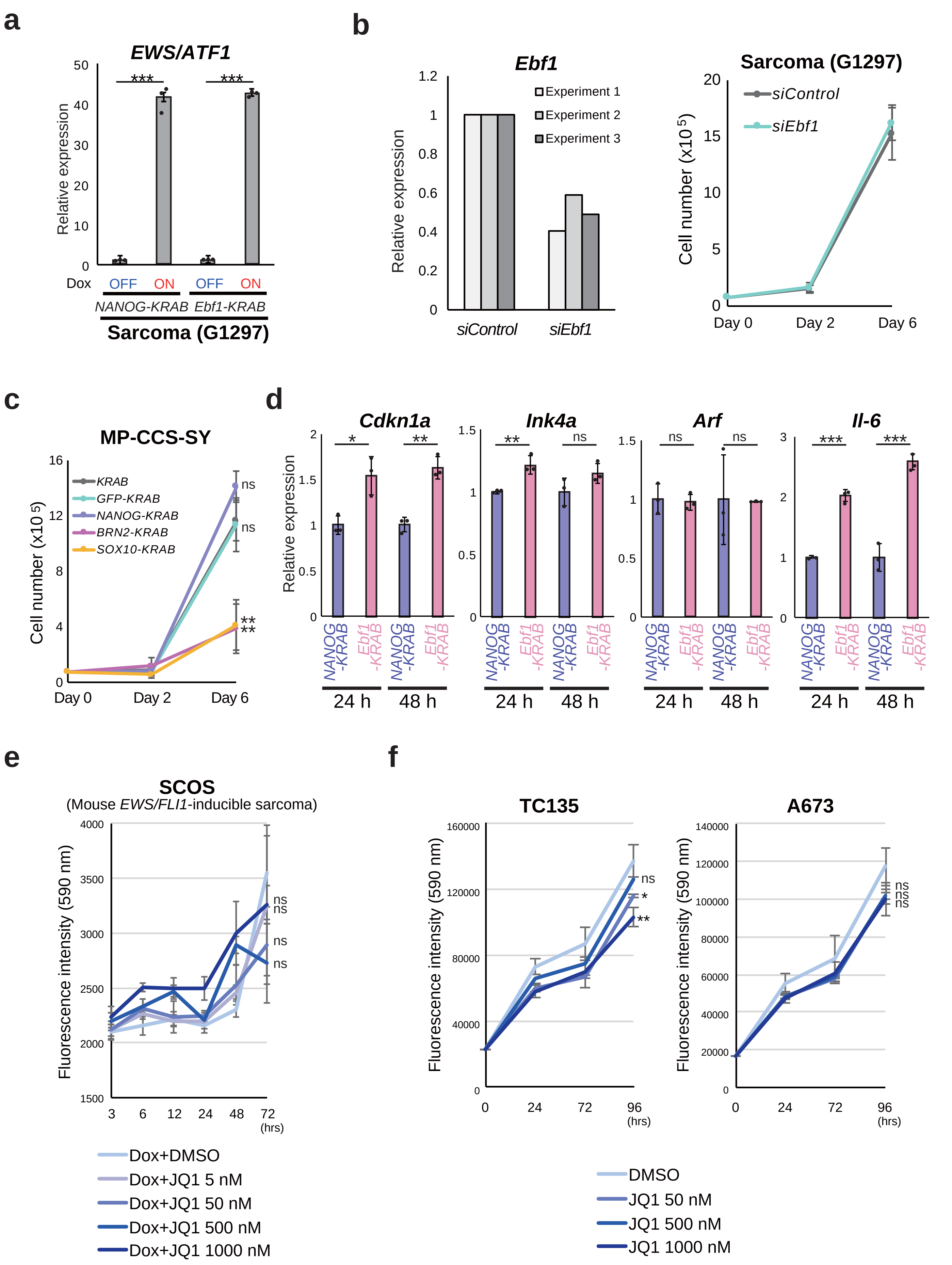

Supplementary Figure 8.
Effect of JQ1 treatment on Ewing sarcoma cell growth

a. Comparison of *EWS/ATF1* expression levels between *Ebf1-KRAB-* and *NANOG-KRAB*-expressing G1297 (***P < 0.0001, two-sided Student's t-test). qRT-PCR reveals that *Ebf1-KRAB* transduction does not reduce *EWS/ATF1* expression in G1297. The induction time of *KRAB* fusion genes is 48 hours. Data are presented as mean ± SD of independent biological triplicates. The expression level of *NANOG-KRAB*-expressing G1297 (Dox0) was set to 1.
b. Knockdown of *Ebf1* does not affect cell growth in G1297. Relative knockdown efficiency is shown in the left panel. Data are presented as mean ± SD of independent biological triplicates.
c. Cell growth assay of *KRAB*-fusion-expressing MP-CCS-SY. *SOX10-KRAB* and *BRN2-KRAB* transduction result in the inhibition of CCS cell growth (ns; not significant, **P < 0.01, two-sided Student's t-test). *KRAB*, *GFP-KRAB*, and *NANOG-KRAB* were used as controls. Data are presented as mean ± SD of independent biological triplicates.
d. A qRT-PCR analysis for *Cdkn1a*, *Ink4a*, *Arf*, and *Il-6* in sarcoma cells (G1297) expressing *NANOG-KRAB* and *Ebf1-KRAB* at 24 h and 48 h. *Ebf1-KRAB*-expressing G1297 exhibits an increased expression of *Cdkn1a* and *Ink4a*, as well as *IL-6* (ns; not significant, *P < 0.05, **P < 0.01, ***P < 0.001, two-sided Student's t-test). Data are presented as the mean ± SD of independent biological triplicates. The mean expression level of *NANOG-KRAB*-expressing G1297 was set to 1.
e. Effect of JQ1 treatment on growth of mouse EWS/FLI1-induced sarcoma cells (ns; not significant, two-sided Student's t-test). Data are presented as mean ± SD of independent biological triplicates.
f. Effect of JQ1 treatment on growth of human Ewing sarcoma cells (ns; not significant, *P < 0.05, **P < 0.01, two-sided Student's t-test). Data are presented as mean ± SD of independent biological triplicates.

Supplementary Figure 9

Figure 4d

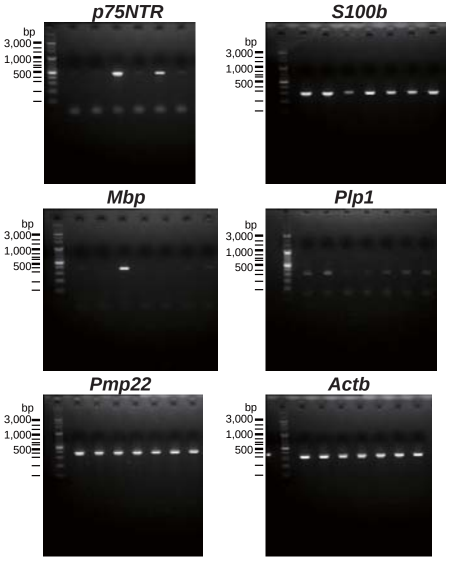

Supplementary Figure 1f

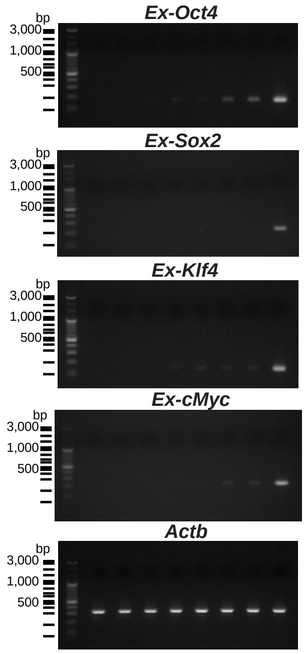

Supplementary Figure 2e

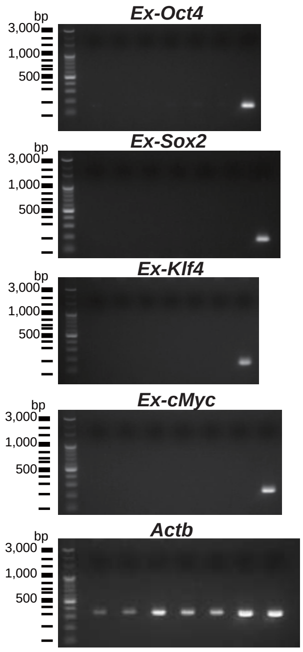

Supplementary Figure 2f

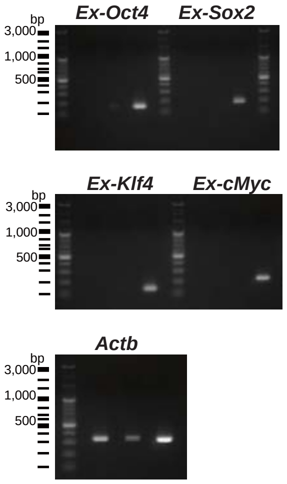

Supplementary Figure 5i

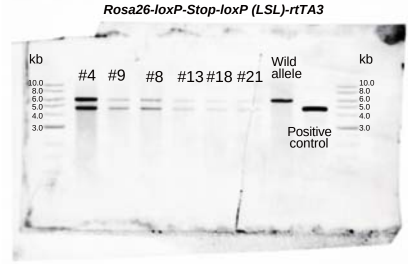

Supplementary Figure 6d

Supplementary Figure 6b

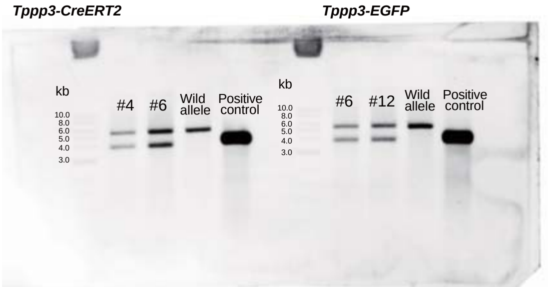

Supplementary Figure 5j

Supplementary Figure 6e

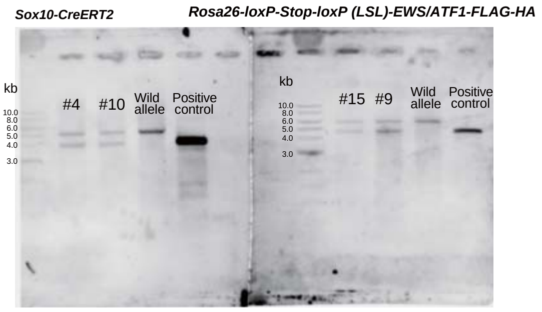

Supplementary Figure 5d

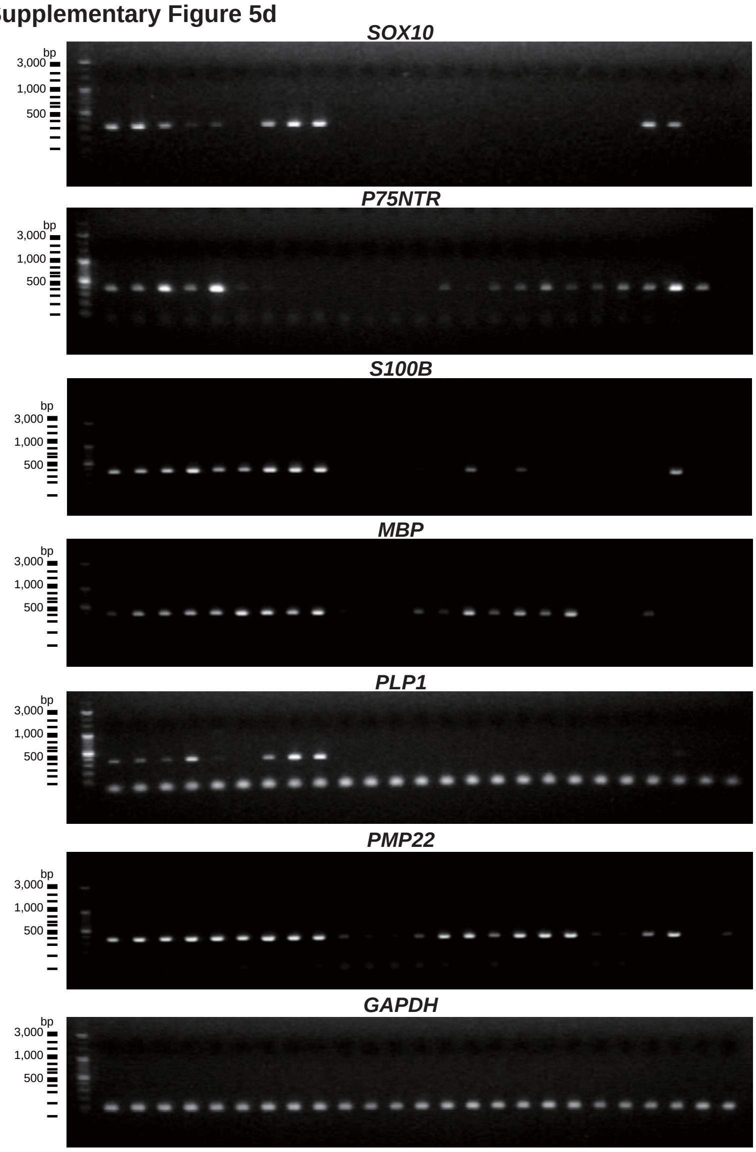

Supplementary Figure 9.
This file contains uncropped gel image data.

Supplementary Table 1: A list of potential mutation sites in sarcoma (G1297) and sarcoma-iPSC #3.

			Base changes		Amino acid changes		Sarcoma (G1297)		Sarcoma-iPSC #3	
Chromosome	position	Gene	Ref.	Alt.	Ref.	Alt.	Mutation freq.	Sequencing depth	Mutation freq.	Sequencing depth
chr1	135,457,866	Nav1	TA	T	Frame shift		0.571428571	21	0.333333333	24
chr2	66,536,265	Scn9a	CAATTCCAGATTAAA	C	Frame shift		0.275862069	29	0.452380952	42
chr2	82,990,281	Fsip2	C	CT	Frame shift		0.462962963	54	0.617021277	47
chr14	30,142,701	Cacna1d	T	A	Stop codon gained		0.489795918	49	0.586206897	58
chr15	91,731,509	Lrrk2	A	T	Stop codon gained		0.355555556	45	0.431372549	51
chr1	9,687,657	Mybl1	T	G	Lys	Thr	0.430232558	86	0.554347826	92
chr1	74,576,049	Zfp142	G	C	Ala	Gly	0.46728972	107	0.467153285	137
chr1	75,175,009	Abcb6	T	G	Glu	Asp	0.417391304	230	0.474358974	312
chr1	92,641,894	Myeov2	C	T	Val	Met	0.392156863	51	0.493975904	83
chr1	154,493,310	Cacna1e	T	C	Asp	Gly	0.43442623	122	0.536585366	123
chr1	166,485,696	Gm4846	T	G	Lys	Thr	0.416666667	96	0.482758621	116
chr1	171,229,915	Fcer1g	G	A	Arg	Trp	0.507042254	71	0.452380952	84
chr1	181,117,449	Nvl	G	A	Thr	Ile	0.453333333	75	0.48	100
chr1	184,727,729	Hlx	T	G	Lys	Thr	0.551724138	29	0.512195122	41
chr2	26,950,603	Stkld1	A	C	Gln	His	0.555555556	45	0.450980392	51
chr2	28,505,328	Gbgt1	G	A	Arg	Gln	0.5	16	0.473684211	38
chr2	60,376,098	Ly75	A	C	Phe	Val	0.466666667	75	0.494736842	95
chr2	90,003,275	Olfr1262	A	G	Lys	Glu	0.365853659	82	0.425	80
chr2	90,003,308	Olfr1262	C	A	His	Asn	0.457627119	59	0.397058824	68
chr2	103,731,413	Nat10	C	G	Gly	Ala	0.469387755	49	0.514285714	70
chr2	122,118,208	Spg11	A	G	Leu	Pro	0.466666667	15	0.421052632	19
chr2	129,075,947	Ttl	G	A	Ala	Thr	0.465116279	43	0.571428571	84
chr2	160,908,453	Emilin3	A	C	Phe	Val	0.313953488	86	0.464285714	112
chr2	163,825,267	Wisp2	T	C	Ser	Pro	0.488372093	43	0.55	60
chr2	174,298,266	Gnas	A	G	Lys	Arg	0.465116279	43	0.465753425	73
chr2	179,935,121	Taf4a	T	C	Ser	Gly	0.3	20	0.466666667	30
chr3	20,228,836	Cpa3	A	C	Val	Gly	0.483870968	62	0.46835443	79
chr3	30,631,424	Lrrc34	G	A	Arg	Cys	1	35	1	43
chr4	21,782,906	Usp45	C	G	Ser	Arg	0.457142857	35	0.588235294	34
chr4	43,415,909	Rusc2	T	C	Leu	Pro	0.5375	80	0.526315789	133
chr4	99,065,385	Dock7	G	C	Ala	Gly	0.515151515	33	0.487179487	39
chr4	114,194,610	Skint11	A	C	Ser	Arg	0.481012658	79	0.461538462	65
chr4	134,202,410	Gm7534	A	C	Leu	Val	0.50390625	256	0.462650602	415
chr4	152,113,859	Plekhg5	T	C	Ser	Pro	0.412698413	63	0.540540541	111
chr4	152,377,037	Chd5	G	C	Asp	His	0.380952381	21	0.68	25
chr5	24,387,499	Atg9b	A	C	Leu	Arg	0.534883721	43	0.538461538	52
chr5	86,197,743	Gnrhr	A	T	Leu	His	0.459459459	37	0.528301887	53
chr5	106,874,850	Hfm1	A	T	Leu	His	0.512195122	41	0.480769231	52
chr5	110,189,798	Golga3	A	C	Asn	His	0.482758621	29	0.468085106	47
chr5	124,401,167	Sbno1	G	C	Ala	Gly	0.426829268	82	0.567010309	97
chr5	125,023,557	Ncor2	T	C	Lys	Arg	0.47826087	138	0.454022989	174
chr5	138,757,965	Fam20c	A	C	Lys	Thr	0.458646617	133	0.431924883	213
chr6	11,987,092	Phf14	A	C	Lys	Thr	0.296875	64	0.362318841	69
chr6	66,637,410	Vmn1r34	A	C	Phe	Val	0.340369393	379	0.362186788	439
chr6	132,312,261	Prpmp5	C	A	Gly	Val	0.277161863	451	0.308712121	528
chr7	10,714,353	Nlrp4b	A	T	Asn	Ile	0.50617284	81	0.422680412	97
chr7	25,638,165	Bckdha	C	G	Ala	Pro	0.49122807	57	0.416666667	96
chr7	34,934,711	Pepd	A	C	Lys	Thr	0.461538462	39	0.407407407	81
chr7	46,701,922	Saal1	A	T	Leu	His	0.426829268	82	0.441489362	188
chr7	83,897,950	Mesdc2	A	C	Lys	Thr	0.354166667	48	0.631578947	57
chr7	86,465,510	Olfr299	A	C	Tyr	Ser	0.397163121	141	0.482517483	143
chr7	102,973,357	Olfr577	C	T	Val	Met	0.525423729	59	0.555555556	63
chr8	107,058,173	Nip7	G	T	Ala	Ser	0.472222222	36	0.463414634	41
chr8	107,413,008	Nob1	G	C	Ser	Trp	0.454545455	22	0.428571429	42
chr8	110,600,251	Hydin	T	G	Phe	Val	0.461538462	65	0.476923077	130
chr8	116,933,299	Cenpn	T	G	Phe	Val	0.46875	32	0.440677966	59
chr9	44,256,464	Nlrx1	T	C	Lys	Arg	0.613636364	44	0.592592593	81
chr9	58,026,196	Cyp11a1	T	G	Phe	Val	0.464285714	56	0.391304348	92
chr9	59,870,963	Myo9a	T	C	Leu	Pro	0.382352941	34	0.538461538	52
chr10	12,720,929	Utrn	T	G	Ser	Arg	0.661654135	133	0.595505618	178
chr10	22,286,289	H60b	A	T	Ser	Cys	0.573529412	68	0.636363636	77
chr10	67,244,839	Jmjd1c	T	C	Ser	Pro	0.483870968	31	0.326530612	49
chr10	75,949,293	Vpreb3	T	C	Leu	Pro	0.568965517	58	0.669724771	109
chr10	79,781,166	Fstl3	T	G	Leu	Arg	0.601123596	178	0.666666667	294
chr10	81,332,714	Tbxa2r	T	G	Val	Gly	0.714285714	56	0.691489362	94
chr10	129,692,035	Olfr803	T	C	Lys	Arg	0.658227848	79	0.783333333	60
chr11	3,819,900	Osbp2	G	C	His	Asp	0.625	56	0.489583333	96
chr11	84,342,011	Acaca	G	A	Glu	Lys	0.558139535	86	0.597826087	92
chr11	87,549,488	Tex14	A	C	Asn	Thr	0.425	80	0.441860465	129
chr11	106,019,137	Kcnh6	T	C	Val	Ala	0.397435897	156	0.553278689	244
chr11	110,244,291	Abca6	A	G	Phe	Leu	0.444444444	36	0.47826087	69
chr11	115,616,648	Slc25a19	T	G	Asn	Thr	0.513513514	37	0.430379747	79
chr12	114,682,664	Ighv1-18	A	T	Ser	Thr	0.50245098	408	0.505102041	392
chr13	22,244,744	Vmn1r194	T	G	Val	Gly	0.388235294	85	0.449438202	89
chr13	74,150,794	Slc9a3	T	G	Phe	Val	0.462121212	132	0.445121951	164
chr13	100,489,143	Gtf2h2	A	T	Leu	His	0.490196078	51	0.512820513	78
chr13	113,095,902	Gzma	T	G	Lys	Thr	0.52173913	69	0.53125	96
chr14	20,651,261	Myoz1	A	G	Phe	Leu	0.428571429	14	0.5	32
chr15	98,457,041	Olfr281	C	T	His	Tyr	0.52238806	67	0.441176471	102
chr15	101,892,462	Krt76	C	A	Cys	Phe	0.390243902	41	0.425	40
chr16	18,437,532	Txnrd2	T	G	Val	Gly	0.48	25	0.421052632	19
chr16	36,969,911	Fbxo40	A	C	Leu	Arg	0.622807018	114	0.555555556	108
chr16	88,627,399	2310079G19Rik	C	G	Cys	Ser	0.476923077	65	0.365079365	63
chr17	24,702,480	Tbl3	T	A	Asp	Val	0.492537313	67	0.476190476	126
chr17	25,111,118	Telo2	T	A	Lys	Met	0.523364486	107	0.549618321	131
chr17	34,696,037	Tnxb	T	C	Ser	Pro	0.478378378	370	0.435763889	576
chr17	40,880,380	9130008F23Rik	A	C	Leu	Val	0.435714286	140	0.475903614	166
chr17	72,205,866	Alk	A	C	Val	Gly	0.40070922	282	0.493112948	363
chr18	6,225,597	Kif5b	T	G	Asn	His	0.432432432	185	0.464435146	239
chr18	36,796,519	Zmat2	T	G	Ser	Ala	0.390243902	41	0.596153846	52
chr18	64,265,605	St8sia3	C	G	Ala	Gly	0.460526316	76	0.474226804	97
chr19	13,145,793	Olfr1459	T	C	Ser	Gly	0.470588235	221	0.488479263	217
chrX	56,960,158	Adgrg4	A	G	Asn	Ser	1	20	1	33
chrX	66,654,905	Slitrk2	A	T	Lys	Met	0.980392157	51	1	70

Supplementary Table 2: Primers used in this study.

	Genes	Forward (5' ⇒ 3')	Reverse (5' ⇒ 3')	Reference
	**Mouse**			
	EWS/ATF1	CAATATAGCCAACAGAGCAGCAG	CTCCAAGGGGAGGACTTTTG	
	Nanog	TGCTTACAAGGGTCTGCTACTG	TAGAAGAATCAGGGCTGCCTTG	
	Oct3/4 (endogenous)	TCCCATGCATTCAAACTGAG	CCACCCCTGTTGTGCTTTTA	
	Ink4a	CCGCTGCAGACAGACTGG	TCGAATCTGCACCGTAGTTG	
	Arf	GTCGCAGGTTCTTGGTCACT	TCGAATCTGCACCGTAGTTG	
	Cdkn1a	TTGCACTCTGGTGTCTGAGC	TCTGCGCTTGGAGTGATAGA	
	IL-6	GTTCTCTGGGAAATCGTGGA	GGTACTCCAGAAGACCAGAGGA	（Mosterio et al. 2016）
	Ebf1	CAGCAGTGAAACAGAAGAGTGC	ACATGGGAGGGACAATCATGC	
	Actb	GCCAACCGTGAAAAGATGAC	TCCGGAGTCCATCACAATG	
	**Human**			
	EWS/ATF1	GAAGAGGGGGATTTGATCGT	TTTTCTGCCCCGTGTATCTT	
	Sox10	GGCTCCCCCATGTCAGAT	GCTGATCTCACCAATGTCCA	
**qRT-PCR**	*MPZ*	GAAATTGCACAAGCCAGGAA	CCTTGGCCTTCTTCTCACTG	
	NGFR	CCCTGTCTATTGCTCCATCC	CTGTTGGCTCCTTGCTTGTT	
	TYR	TTCATCCAAAGATCTGGGCTA	CAGATCCGACTCGCTTGTTC	
	MITF	GAAATCTTGGGCTTGATGGA	GGACAGGAGTTGCTGATGGT	
	DCT	ACAAACGCTTTGCCACATTC	AGGCATCTGCAGGAGGATTA	
	S100B	AGGGAGACAAGCACAAGCTG	TCCACAACCTCCTGCTCTTT	
	EGR1	AGCCCTACGAGCACCTGAC	AGCGGCCAGTATAGGTGATG	
	EGR2	GGTGACCATCTTTCCCAATG	AGGGTCAATGGAGAACTTGC	
	EGR3	CAGCGACTCGGTAGTCCATT	GGAAGGAGCCGGAGTAAGAG	
	TPPP3	ATGGCAAGAACTGGGCCAAG	TTGATGACCCGAGCAGACTTC	
	PLP1	CCAAATGACCTTCCACCTGT	CATGAGTTTAAGGACGGCAAA	
	PMP22	CCTCAGGAAATGTCCACCAC	AGGGTGAAGAGTTGGCAGAA	
	MBP	ACGCAGGCAAACGAGAATTA	CGTGAAAGTTCACCCAGGTT	
	GAPDH	ATGGGGAAGGTGAAGGTCG	GGGGTCATTGATGGCAACAATA	
	**Mouse**			
	p75NTR	CCTGCCAGGACAAACAGAAC	GCAGTTTCTCTCCCTCTGGT	
	Mbp	GGCTGGAAAGAAGAGAAGCG	TGGCATGGTCCATGGTACTT	
	Plp1	TGTTGTATGGCTCCTGGTGT	CCATGAGTTTAAGGACGGCG	
	S100b	CCCAGTTCTCTCTGCAGGAA	CTGGAAGTCACACTCCCCAT	
	Pmp22	TTGCTCTTCGTCTCCACCAT	ATGATACCACTGAGGAGGGC	
	Actb	GCTACAGCTTCACCACCACA	CTTCTGCATCCTGTCAGCAA	
	**Human**			
**RT-PCR**	*SOX10*	CTACAAGAGCGCCCACTTGG	GTAGCTGCTCACATGGCCTG	
	P75NTR	CCGACAACCTCATCCCTGTC	CTATGTGCTCGGGCTGGTAG	
	S100B	ATGTCTGAGCTGGAGAAGGC	AGCTCCTACTAGGCTGCAAG	
	MBP	CACGGACAACCTCAGAGGAC	CTCTGTGCCTTGGGAGGAAG	
	PLP1	GTGGCCACTGGATTGTGTTT	AAACAATGACACACCCGCTC	
	PMP22	GCTCCTCCTGTTGCTGAGTA	ATCCGAGTTGAGATGCCACT	
	Ex-hOCT3/4	GCTCTCCCATGCATTCAAACTGA	CTTACGCGAAATACGGGCAGACA	
	Ex-hSOX2	TTCACATGTCCCAGCACTACCAGA	GACATGGCCTGCCCGGTTATTATT	
	Ex-hKLF4	CCACCTCGCCTTACACATGAAGA	GACATGGCCTGCCCGGTTATTATT	
	Ex-h-cMYC	ATACATCCTGTCCGTCCAAGCAGA	GACATGGCCTGCCCGGTTATTATT	
**Bisulfite genomic sequence**	*Nanog* promoter	GATTTTGTAGGTGGGATTAATTGTGAATTT	ACCAAAAAAACCCACACTCATATCAATATA	（Takahashi and Yamanaka 2006）
	*Oct3/*4 distal enhancer	GGTTTTAGAGGTTGGTTTTGGG	CATCTCTCTAACCCTCTCCATAAATC	（Theunissen, Costa et al. 2011）
