# Supplementary material for: Cell-type dependent enhancer binding of the EWS/ATF1 fusion gene in clear cell sarcomas
Source: Nat Commun. 2019 Sep 5;10:3999. doi: 10.1038/s41467-019-11745-1 (PMC6728361; doi:10.1038/s41467-019-11745-1)
Supplement: Supplementary file 2 — Reporting Summary [file 41467_2019_11745_MOESM2_ESM.pdf]

nature research

Corresponding author(s): Yasuhiro Yamada

Last updated by author(s): Jul 19, 2019

Reporting Summary

Nature Research wishes to improve the reproducibility of the work that we publish. This form provides structure for consistency and transparency in reporting. For further information on Nature Research policies, see Authors & Referees and the Editorial Policy Checklist.

Statistics

For all statistical analyses, confirm that the following items are present in the figure legend, table legend, main text, or Methods section.

n/a	Confirmed	
☐	☒	The exact sample size (*n*) for each experimental group/condition, given as a discrete number and unit of measurement
☐	☒	A statement on whether measurements were taken from distinct samples or whether the same sample was measured repeatedly
☐	☒	The statistical test(s) used AND whether they are one- or two-sided<br>*Only common tests should be described solely by name; describe more complex techniques in the Methods section.*
☒	☐	A description of all covariates tested
☒	☐	A description of any assumptions or corrections, such as tests of normality and adjustment for multiple comparisons
☐	☒	A full description of the statistical parameters including central tendency (e.g. means) or other basic estimates (e.g. regression coefficient) AND variation (e.g. standard deviation) or associated estimates of uncertainty (e.g. confidence intervals)
☒	☐	For null hypothesis testing, the test statistic (e.g. *F*, *t*, *r*) with confidence intervals, effect sizes, degrees of freedom and *P* value noted<br>*Give P values as exact values whenever suitable.*
☒	☐	For Bayesian analysis, information on the choice of priors and Markov chain Monte Carlo settings
☒	☐	For hierarchical and complex designs, identification of the appropriate level for tests and full reporting of outcomes
☐	☒	Estimates of effect sizes (e.g. Cohen's *d*, Pearson's *r*), indicating how they were calculated

Our web collection on statistics for biologists contains articles on many of the points above.

Software and code

Policy information about availability of computer code

Data collection	Prism7 (Graphpad Software), Genespring version 14.8 (Agilent Technologies),R;
Data analysis	*Provide a description of all commercial, open source and custom code used to analyse the data in this study, specifying the version used OR state that no software was used.*

For manuscripts utilizing custom algorithms or software that are central to the research but not yet described in published literature, software must be made available to editors/reviewers. We strongly encourage code deposition in a community repository (e.g. GitHub). See the Nature Research guidelines for submitting code & software for further information.

Data

Policy information about availability of data

All manuscripts must include a data availability statement. This statement should provide the following information, where applicable:

- Accession codes, unique identifiers, or web links for publicly available datasets
- A list of figures that have associated raw data
- A description of any restrictions on data availability

GSE124873

Field-specific reporting

Please select the one below that is the best fit for your research. If you are not sure, read the appropriate sections before making your selection.

☒ Life sciences ☐ Behavioural & social sciences ☐ Ecological, evolutionary & environmental sciences

Life sciences study design

All studies must disclose on these points even when the disclosure is negative.

Sample size	Sample size is described in experimental materials and methods section, figures or figure legends. RT-qPCR experiments were performed in triplicate (Figures 3c, 3f, 5b, S4b, S4f, S5g, S8a, S8d) and duplicate (Figure S4e). To confirm difference of Oct3/4 and Nanog expression level between established iPSC clones, the results from 6 (Figure S1b) and 3 (Figure S2b, S2c) independent clones were listed. RT-PCR experiments were performed in 7 (Figure 4d) or 6 (Figure S1f) or 5 (Figure S2e) independent clones. More than 6 (Figures 1h, 1i, 4i, 6b, 6e) and independent mice were collected to ensure statistical power of detection. Microarray was performed in 1 or 2 sample in each condition (Figure 4e, 7a, S5f). To confirm knockdown efficiency, RT-qPCR was performed in 1 sample in which was used for microarray (Figure S5e). To confirm difference of global gene expression between established iPSC clones, microarray was performed in 2 independent iPSCs derived from same sarcoma cell line (G1297) (Figure S1C). ChIPseq data were obtained from 2 sample in each condition (Figures 7b, 7c, 7d, 7f, S7b, S7c, S7d, S7e, S7f). Representative heat map results from one of two ChIP seq data listed in Figure S7g. Bisulfite sequencing was performed at least 7 replicate clones in each cell types. With respect to Sarcoma-iPSC, more than 2 independent clones derived from different sarcoma cell line (G1297 and K11) were used (Figure 1e-1i). Cell growth assay was performed in 3 (Figure 3a, 7e, 7h, 7i, S8b, S8c, S8e, S8f) or 2 (Figure 3d) samples in each condition. Array CGH analysis was performed in 1 sample in each conditions (Figure 1b, S1g, S4a). Exome analysis and direct sequencing were performed 1 sample in each cell types (Figure 1c, S1h, S4a). SA-β-gal positive cell ratio was calculated from more than 3 are from identical cell culture condition (Figure 3b and 3e, 7g). For MP-CCS-SY xenograft studies, the size of more than 12 clumps were calculated in each condition (Figure 7j).
Data exclusions	Since we used chimeric mice to evaluate pathological data, we excluded mice with no chimeric contribution. We also excluded a few mice which unexpectedly died during Dox treatment. We didn't excluded any other data from the analysis to avoid the arbitrary selection.
Replication	Repeating experiments demonstrated that our findings are constantly reproducible. See experimental materials and methods section.
Randomization	All samples (germline transmitted mice, CCS cell line, CCS-iPSC MEFs, CCS-iPSC, Cotrol-iPSC and human CCS cell lines) were allocated at random. Chimeric mice used for pathological experiment were allocated to arrange coat color chimerism to avoid the effect of chimerism (See Table1).
Blinding	We used blinding for MP-CCS-SY xenograft studies (Figure 7j) and cell count for SA-β-gal positive cell ratio (Figure 3b, 3e, 7g).

Reporting for specific materials, systems and methods

We require information from authors about some types of materials, experimental systems and methods used in many studies. Here, indicate whether each material, system or method listed is relevant to your study. If you are not sure if a list item applies to your research, read the appropriate section before selecting a response.

Materials & experimental systems

n/a	Involved in the study	
☐	☒	Antibodies
☐	☒	Eukaryotic cell lines
☒	☐	Palaeontology
☐	☒	Animals and other organisms
☒	☐	Human research participants
☒	☐	Clinical data

Methods

n/a	Involved in the study	
☐	☒	ChIP-seq
☒	☐	Flow cytometry
☒	☐	MRI-based neuroimaging

Antibodies

Antibodies used	All supplier names, catalog numbers, clone names of primary antibodies which we used in this study were described in experimental materials and methods section.
Validation	Validation of dilution was determined in accordance with manufacturer's directions. In some primary antibodies, we optimized the dilution to fit our staining protocols.

Eukaryotic cell lines

Policy information about cell lines

Cell line source(s)	Mouse clear cell sarcoma cell line was established from EWS/ATF1-inducible CCS model. CCS-iPSCs were generated from the CCS cell line. CCS-iPSC MEFs were generated from chimeric mice derived from CCS-iPSCs. ETF-iPSCs were generated from ear tip fibroblasts derived from CCS model. EWS/ATF1-inducible control ESCs were generated from KH2 ESCs. Human CCS cell lines (KAS, MP-CCS-SY) were kindly provided form T. Nakamura and H. Moritake, respectively and Ewing sarcoma cell lines (TC135 and A673) are commercially available. Mouse Ewing sarcoma cell line (SCOS) was established from Ewing sarcoma

	model.
Authentication	Authentication of each cell lines were performed with genotype PCR and genome sequence.
Mycoplasma contamination	Some cell lines were obtained from SPF mice. Human CCS cell lines were certified no mycoplasma contamination before they were injected into nude mice.
Commonly misidentified lines (See ICLAC register)	N/A

Animals and other organisms

Policy information about studies involving animals; ARRIVE guidelines recommended for reporting animal research

Laboratory animals	Germline transmited mice used in this study were maintained in C57/BL6 or mixed (C57/BL6 and 129) background. We used 4-week-old mice and chimeric mice to treat with Dox. In pathological investigations, we used both male and female mice.
Wild animals	N/A
Field-collected samples	N/A
Ethics oversight	CiRA Animal Experiment Committee

Note that full information on the approval of the study protocol must also be provided in the manuscript.

ChIP-seq

Data deposition

☒ Confirm that both raw and final processed data have been deposited in a public database such as GEO.

☒ Confirm that you have deposited or provided access to graph files (e.g. BED files) for the called peaks.

Data access links<br>*May remain private before publication.*	GSE124873
Files in database submission	G1297_Dox On 12h_HA<br>G1297_Dox On 12h_Input<br>H3K27ac_G1297_DOX_Off<br>H3K27ac_Input_DOX_Off<br>MEF_Dox On 12h_HA<br>MEF_Dox On 12h_Input<br>G1297_ON_Input<br>G1297_Ebf1KRAB_48h<br>G1297_NanogKRAB_48h
Genome browser session (e.g. UCSC)	N/A

Methodology

Replicates	ChIP-seq data were obtained from 2 sample in each condition
Sequencing depth	G1297_12h_HA.fastq 27,393,111 145 bp<br>G1297_12h_INT.fastq 22,108,435 145 bp<br>H3K27ac_G1297_DOX_off.fastq 24,340,998 101 bp<br>H3K27ac_Input_DOX_off.fastq 48,072,211 101 bp<br>MEF_12h_HA_R1.fastq 27,393,111 145 bp<br>MEF_12h_INT_R1.fastq 26,375,781 145 bp<br>G1297_ON_Input 42,857,582 101 bp<br>G1297_Ebf1KRAB_48h 66,188,950 109 bp<br>G1297_NanogKRAB_48h 68,077,469 109 bp
Antibodies	Mouse monoclonal anti-acetyl Histone H3 (Lys27), Monoclonal Antibody<br>Lab, 308-34843<br>Mouse IgG2a Isotype Control-ChIP Grade, Abcam, ab18413<br>anti HA antibody(Monoclonal Mouse IgG 1-κ):nacalai cat# 06340-54<br>Lot#M4H0340 .
Peak calling parameters	Peaks were called using MACS v1.4.2 to identify regions of ChIP/FAIRE-seq peaks over background with p value = 1×10-9
Data quality	FastQC was used to ensure the raw data qualities.

Software

We analyzed ChIP/FAIRE-seq data by mapping the reads using Bowtie2. The sequencing reads were aligned to mouse genome build mm9. We used the MACS version 1.4.2 peak finding algorithm to identify regions of ChIP/FAIRE-seq enrichment over background with p value = 1×10-9. Super enhancers were identified by H3K27ac with ROSE pipelines. To analyze and visualize the mapped reads, ngsplot was used. The motif analysis was performed using HOMER (Hypergeometric Optimization of Motif EnRichment) software.
